# Multiplex three-dimensional optical mapping of tumor immune microenvironment

**DOI:** 10.1038/s41598-017-16987-x

**Published:** 2017-12-05

**Authors:** Steve Seung-Young Lee, Vytautas P. Bindokas, Stephen J. Kron

**Affiliations:** 1 Department of Molecular Genetics and Cell Biology, The University of Chicago, Chicago, IL USA; 2 Ludwig Center for Metastasis Research, The University of Chicago, Chicago, IL USA; 3 Integrated Light Microscopy Facility, The University of Chicago, Chicago, IL USA

## Abstract

Recent developments in optical tissue clearing and microscopic imaging have advanced three-dimensional (3D) visualization of intact tissues and organs at high resolution. However, to expand applications to oncology, critical limitations of current methods must be addressed. Here we describe transparent tissue tomography (T3) as a tool for rapid, three-dimensional, multiplexed immunofluorescent tumor imaging. Cutting tumors into sub-millimeter macrosections enables simple and rapid immunofluorescence staining, optical clearing, and confocal microscope imaging. Registering and fusing macrosection images yields high resolution 3D maps of multiple tumor microenvironment components and biomarkers throughout a tumor. The 3D maps can be quantitatively evaluated by automated image analysis. As an application of T3, 3D mapping and analysis revealed a heterogeneous distribution of programmed death-ligand 1 (PD-L1) in Her2 transgenic mouse mammary tumors, with high expression limited to tumor cells at the periphery and to CD31^+^ vascular endothelium in the core. Also, strong spatial correlation between CD45^+^ immune cell distribution and PD-L1 expression was revealed by T3 analysis of the whole tumors. Our results demonstrate that a tomographic approach offers simple and rapid access to high-resolution three-dimensional maps of the tumor immune microenvironment, offering a new tool to examine tumor heterogeneity.

## Introduction

Malignant tumors can be considered abnormal organs that arise as proliferating cancer cells recruit and subvert host vasculature, immune cells and fibroblasts to form a supportive stroma, providing the means to continue growth and to displace and invade normal tissue^1^. In turn, tumor stroma and the extracellular matrix and other components of the interstitium can significantly influence therapeutic response by affecting drug delivery, supporting tumor cell survival and suppressing anti-tumor immune response^2,3^. All of these factors mediate their effects within the context of the complex, three-dimensional architecture of the tumor microenvironment, characterized by a heterogeneous distribution of cells and local features such as hypoxic regions, lymphocytic infiltration, and pushing margins that may all have functional significance^4^. As such, there is considerable interest in new approaches to 3D analysis of the tumor microenvironment toward predicting and monitoring response to therapy^5^.

Immunolocalization of protein biomarkers in thin tissue sections by chromogenic immunohistochemistry (IHC) is a well-validated and broadly used clinical assay to examine a wide range of cancer and stromal antigens, including immune biomarkers such as checkpoint blockade targets. While the initial use of immunofluorescence (IF) to examine tissue long precedes IHC^6^, IF is rarely used for biomarker analysis in cancer specimens due to high nonspecific background and low sensitivity compared to IHC. However, there is renewed interest in fluorescence detection based on the potential for multiplex immunodetection to reveal complex features of the tumor microenvironment such as anti-tumor immune responses^7^.

A common drawback of 2D methods is that they are highly subject to the confounding effects of heterogeneity, which has particular relevance in evaluating the tumor microenvironment. That both IHC and IF rely on imaging in thin tissue sections complicates their application to comprehensive, 3D mapping in tumors. While 3D reconstruction from serial thin sections can yield high resolution 3D maps^8,9^, current tomographic methods remain poorly matched to the demands of the clinical environment, where speed is at a premium and simple, robust methods are required.

Up until recently, a lack of practical methods for 3D tissue analysis had similarly limited progress in analysis of neural connectivity in the central nervous system. However, advances in tissue optical clearing, fluorescent staining and high resolution 3D microscopic imaging of brain tissue have made significant impacts in neuroscience^10–13^. Robust methods such as CLARITY^14^ enable mapping the distributions of multiple antigens in intact mouse brains at sub-cellular resolution, allowing reliable tracing of connections over centimeter distances. The same methods for tissue clearing, immunostaining and 3D imaging strategies are currently being applied to tumor tissue, yielding compelling results^15–22^. At the same, this work has also revealed fundamental limitations of CLARITY and related approaches including tissue distortion, antigen loss, uneven immunostaining, limited imaging depth and low sample throughput^23^.

To address these challenges, we have developed Transparent Tissue Tomography (T3) in which lightly fixed tumors are cut into thick sections that are immunostained with fluorescently labeled primary antibodies, optically cleared, and imaged by confocal microscopy^24–26^. Via image processing and tomographic reconstruction, T3 delivers a 3D model of the distribution of multiple antigens through the whole mouse tumor. Thus, T3 enables visualization and analysis of patterns of expression of multiple tumor microenvironment biomarkers at microscopic, mesoscopic and macroscopic scales. Here, we demonstrate the value of T3 immunostaining, tissue clearing, and 3D imaging by mapping the pattern of expression of an immune checkpoint regulator, programmed cell death protein-ligand 1 (PD-L1), by both tumor and stromal cells in spontaneous mammary tumors formed in an MMTV-Her2/neu transgenic mouse model. We confirm and extend prior observations regarding the heterogeneous distribution of PD-L1 in tumors and discover a new pattern of expression in tumor vasculature. This work establishes T3 as a novel tool for high resolution, quantitative 3D analysis of the tumor immune microenvironment.

## Results

### Transparent Tissue Tomography (T3)

**Figure 1:**
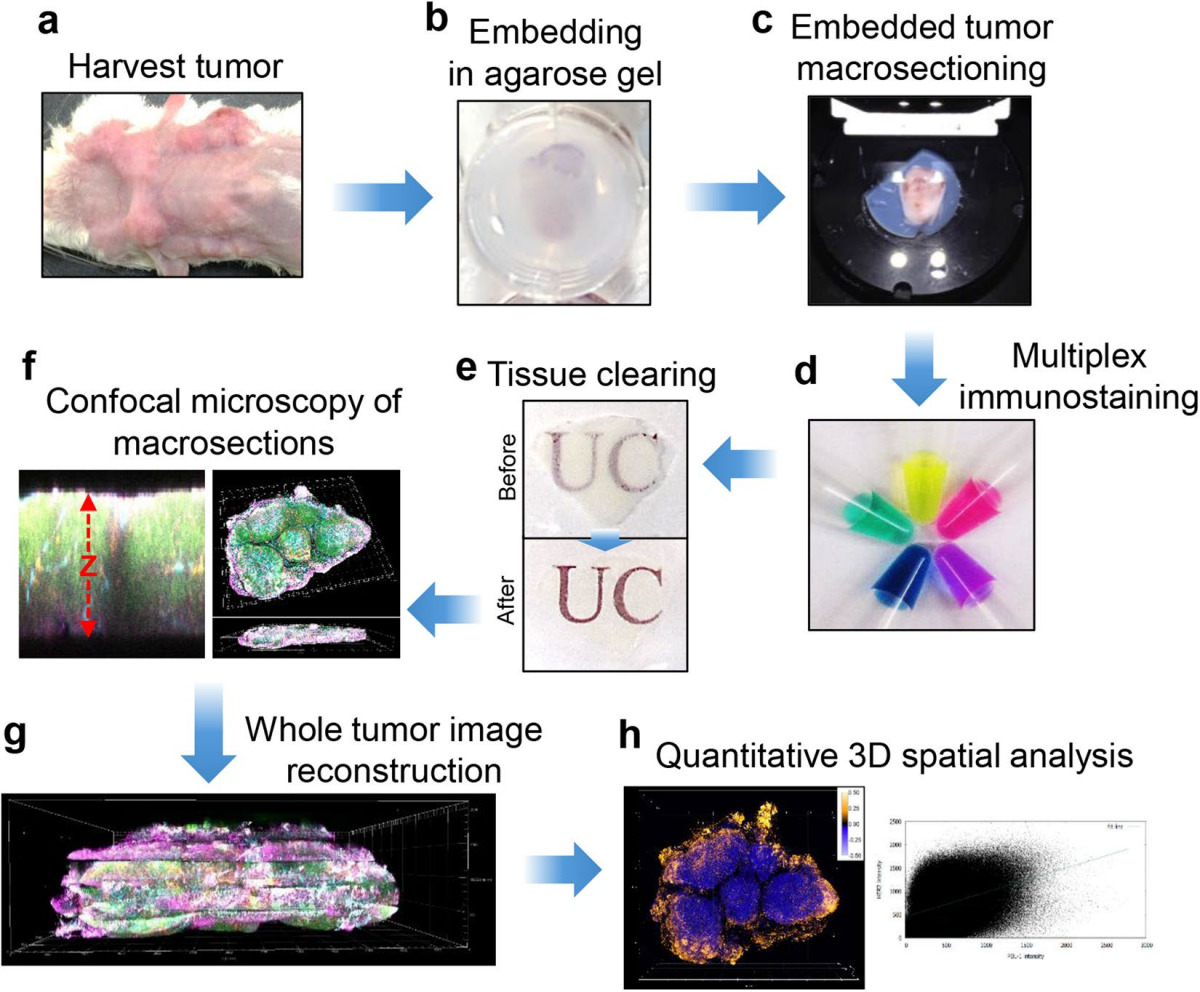
Workflow of transparent tissue tomography (T3) for whole mouse tumors. (**a**) Tumors were excised from a mammary gland of a BALB-NeuT mouse. (**b**) The tumors were fixed with 2% paraformaldehyde solution following by embedding into 2% agarose gel. (**c**) The embedded tumors were sectioned at 400 μm thickness using a vibratome. (**d**) Collected tumor macrosections were stained with fluorescent primary antibodies. (**e**) After washing and fixing, the macrosections were optically cleared by incubating with D-fructose solutions. (**f**) Immunostaining completion for macrosection was confirmed by optical cross-section (Z) scanning using confocal microscopy, and then each macrosection was imaged by XYZ scanning with multi-excitation and emission filters. (**g**) Whole tumor image was reconstructed by concatenating macrosection images, and (**h**) Quantitative 3D spatial analysis of tumor microenvironmental components and biomarkers in the whole tumor was performed using Fiji software.

To model analysis of microenvironmental heterogeneity in human breast tumors, we examined expression of cancer cell and stromal biomarkers in spontaneous mammary tumors expressing rat Her2 formed in female BALB-NeuT mice^27^. We excised and lightly fixed tumors, cast them in agarose and cut <1 mm thick macrosections by vibratome. Processing the tissue in sections facilitated the critical steps of optical clearing, immunofluorescence staining, and confocal imaging (Fig. 1, Table 1). Considering previously reported tissue clearing agents, D-fructose appeared particularly promising based on its ability to achieve brain and skin transparency with minimal tissue volume change by simple immersion for *ex vivo* and *in vivo* deep tissue imaging^28,29^. For tumor macrosections, transparency was increased by equilibration with higher concentrations of D-fructose (Supplementary Fig. S1). We determined that a satisfactory level of transparency for imaging could be achieved by incubating 400 µm thick tumor macrosections in 80% (w/v) D-fructose solution (Fig. 1e). Fluorescent primary antibodies were prepared by conjugating amine-reactive fluorophores (N-hydroxysuccinimide (NHS)-esters) at specific dye:antibody molar ratios (Supplementary Table S1). By obviating the need for secondary antibodies, direct labeling enabled simultaneous staining with and independent detection of multiple primary antibodies. Antibody penetration into tumor macrosections was determined by incubation with fluorescent anti-Her2 antibody at 4 °C (Supplementary Fig. S2a). Cross-section (Z) scanning of tumor macrosections showed that fluorescent anti-Her2 antibody fully penetrated a 400 μm macrosection after 11 h incubation. When 400 μm macrosections were incubated with five directly labeled fluorescent antibodies, DyLight488-anti-Her2, DyLight550-anti-CD45, DyLight594-anti-Ki-67, DyLight633-anti-CD31, and DyLight680-anti-PD-L1 antibodies, for 18 h and then for 1 h each in 20, 50, and 80% D-fructose solutions, confocal microscopy with a 10X objective confirmed full-thickness staining for each marker (Supplementary Fig. S2b), allowing visualization of the distribution of each antigen through whole macrosections (Supplementary Fig. S3). For automated image processing and quantitative 3D spatial analysis, we developed macros based on the open source image analysis software Fiji^30^ and its plugins (Supplementary Table S2). To reconstruct whole tumors, the digital images of each macrosection were stitched, registered and annealed using the macros. The biomarker expression patterns and their spatial relationships could then be mapped three-dimensionally and quantitatively analyzed in whole tumors. T3 analysis for a whole BALB-NeuT mammary tumor could be completed within 3 days.

**Table 1:** Overview of T3 procedure and average times required for each step.

**Process**	**Average Time**	**Notes**
Fixing, embedding, and macrosectioning	1 h	-Fixing tumor in 2% paraformaldehyde for 10 min
		-Embedding tumor in 2% agarose gel
		-Macrosectioning at 400 μm thickness
		-Fixing macrosections in 2% paraformaldehyde for 5 min
Immunofluorescence staining	18 h	-Incubating macrosections with 5 different fluorescent primary antibodies at 4 °C
Washing and fixing	40 min	-Washing macrosections with PBS × 3 times, and fixing in 2% paraformaldehyde for 10 min
Tissue clearing	3 h	-Incubating macrosections sequentially for 1 h each in 20%, 50%, 80% D-fructose solutions (containing 0.3% (v/v) α-thioglycerol) at 25 °C
Confocal imaging	1 h/section	-Mounting macrosections between coverslips
		-Scanning full volume (6 × 5 × 0.4 mm^3^) with 10X objective (X/Y/Z grid resolutions = 1.78/1.78/6.25 μm), using 5 different excitation and emission settings
Image processing and analysis	1 day	-Using Fiji software and created macros for 3D image reconstruction and analysis

### 3D visualization of multiple tumor microenvironment components and biomarkers in a whole mouse tumor

With the protocol established, we applied T3 to explore spatial patterning of PD-L1 expression within BALB-NeuT mammary tumors. Substantial data provide evidence that cancer cells exploit the programmed cell death receptor-1/ligand-1 (PD-1/PD-L1) pathway to evade immune surveillance^31–33^. Although immunohistochemistry (IHC) analysis of PD-L1 expression in tumor tissue has been approved as a companion test for each of the five therapeutic antibodies for PD-1/PD-L1 immune checkpoint blockade, heterogeneity of PD-L1 expression contributes to uncertainty in determining the PD-L1 status of patient tumors^34–36^. Another challenge is that PD-L1 is expressed not only by tumor cells but also by components of the heterogeneous tumor microenvironment including myeloid cells and T cells, each with different functional consequences^37,38^. As such, fully defining the pattern of PD-L1 expression might deliver a better predictive test for PD-1/PD-L1 blockade therapies.

**Figure 2:**
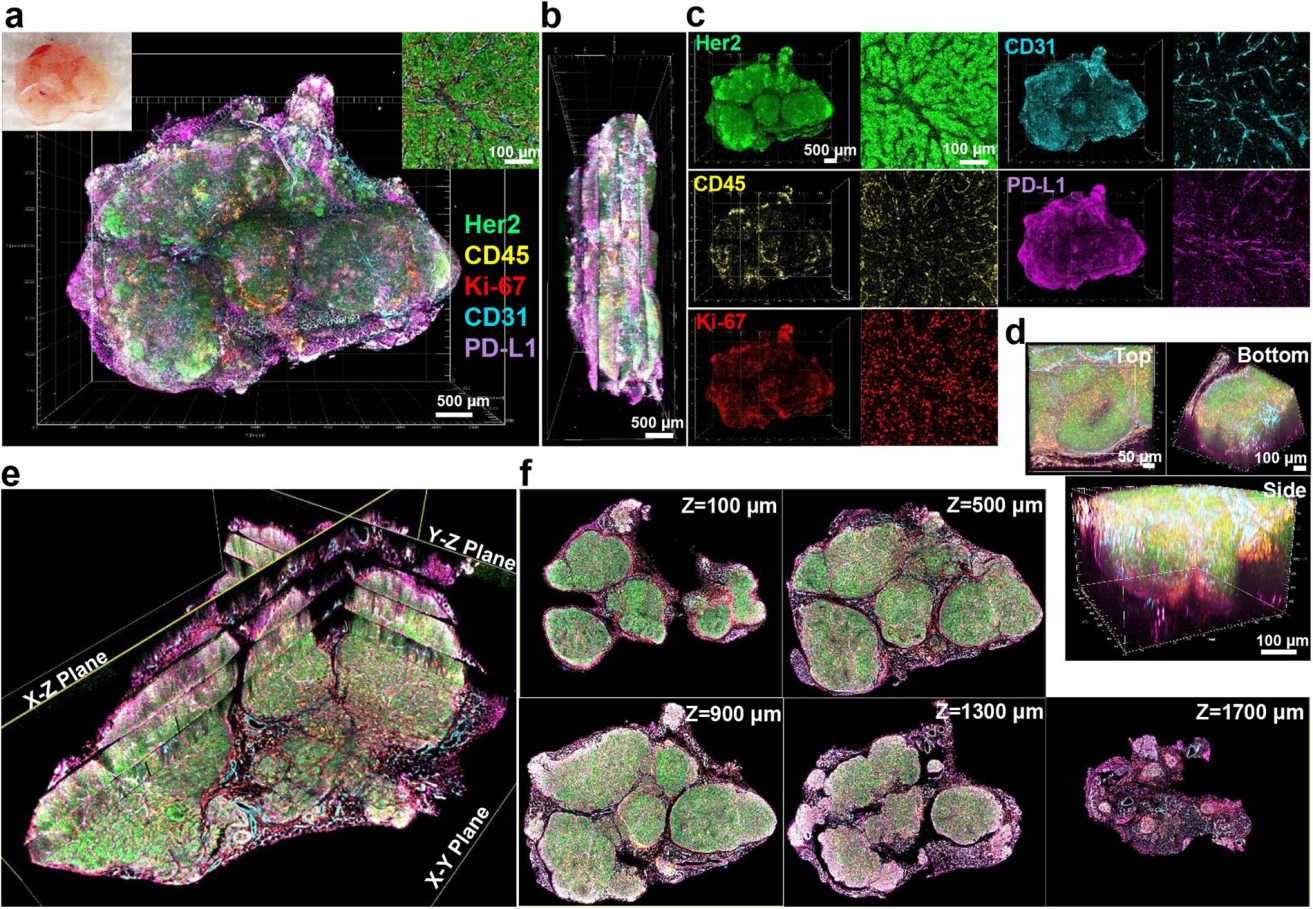
3D mapping of multiple tumor microenvironment components and biomarkers in a whole mouse mammary tumor. (**a**) 3D rendering of reconstructed tumor mass excised from the mammary gland of a BALB-NeuT mouse obtained by fusing images of five 400 μm macrosections each immunostained for Her2 (green), CD45 (yellow), Ki-67 (red), CD31 (cyan), and PD-L1 (magenta). Scale bar: 500 μm. Left top insert shows tumor tissue prior to macrosectioning. Right top insert displays representative 2D image of immunostaining. Scale bar: 100 μm. (**b**) Lateral view of reconstructed tumor. Scale bar: 500 μm. (**c**) 3D (left) and 2D (right) channel images for each marker, showing distinct patterns of expression of the cellular markers and biomarkers across tumor. Scale bars: 500 μm (left), 100 μm (right). (**d**) Distinct views of a volume within the rendered tumor model (580 × 580 × 400 μm). Scale: 50 μm (left), 100 μm (right, bottom). (**e**) Tomographic visualization of the reconstructed tumor image with multiple orthogonal planes (X-Y, X-Z, Y-Z planes). (**f**) Serial tomographic sections of X-Y planes at different Z-stack depths (100, 500, 900, 1300, and 1700 μm).

To examine heterogeneity across whole tumors, we took advantage of the multifocal nature of autochthonous tumors in mammary glands in the BALB-NeuT mouse, a BALB/c genetically engineered mouse (GEM) model that expresses rat Her2/neu under the MMTV promoter^39^. Initially, we examined a fused mass apparently composed of multiple malignant foci and compared it to tumors that appeared to have arisen from a single focus. By applying the T3 protocol to macrosections of each tumor and then registering and fusing each macrosection image, we mapped expression of PD-L1, the tumor marker Her2, the immune cell marker CD45, the cell proliferation marker Ki-67, and the endothelial marker CD31 (Fig. 2a–c, Supplementary Video 1, and Supplementary Fig. S4). By zooming in on specific regions, T3 revealed the distributions of biomarkers at cellular resolution (Fig. 2d and Supplementary Video 2). Given the volumetric data on multiple markers, it was also possible to examine virtual sections (Fig. 2e and Supplementary Video 3) and projections (Fig. 2f and Supplementary Video 4), which provide 2D representations of the data, facilitating evaluation of distributions of biomarkers and their spatial relationships.

### 3D mapping of PD-L1 immunoreactivity of Her2 positive cancer cells

**Figure 3:**
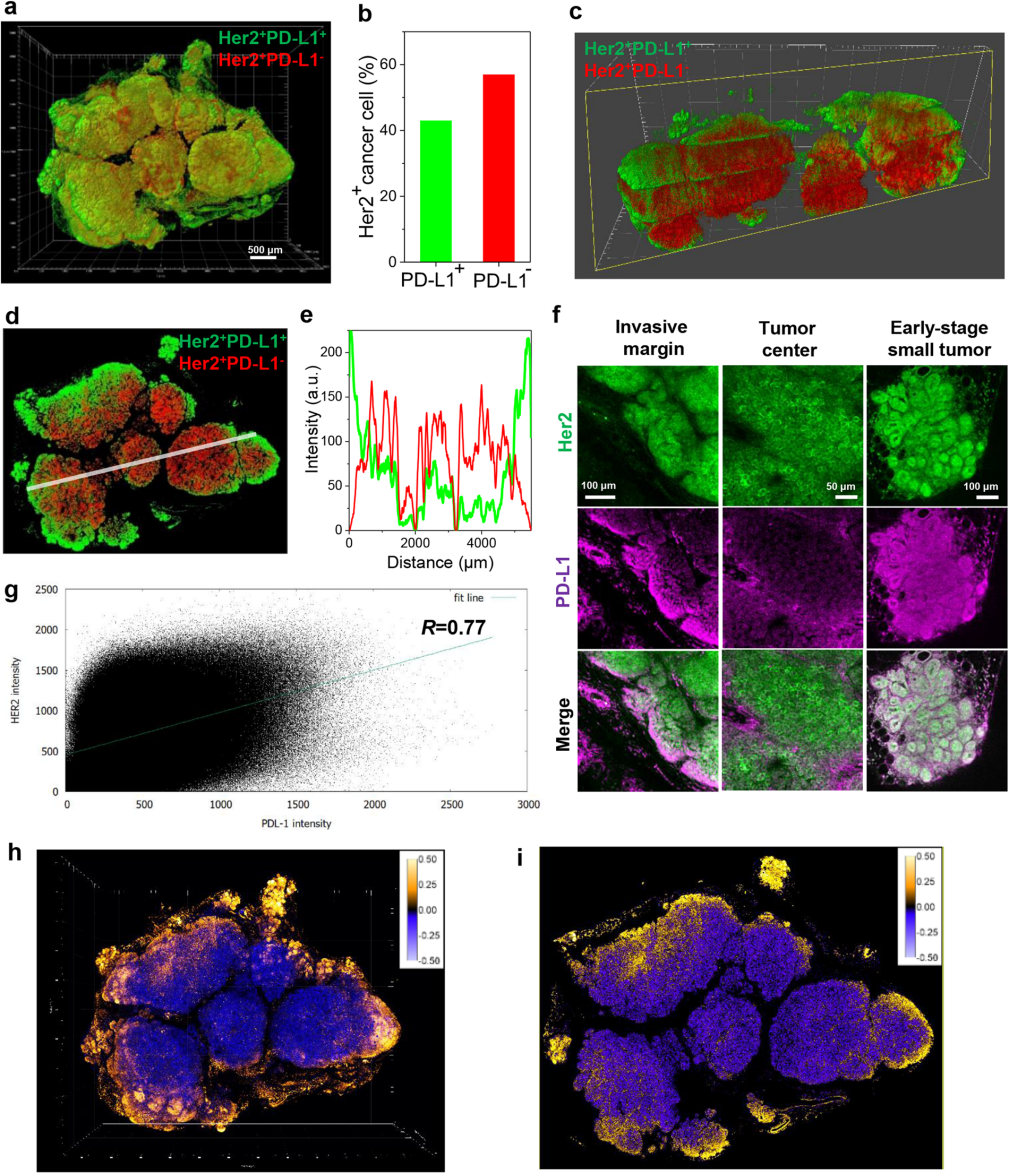
Spatial mapping and analysis of Her2 and PD-L1 expression. (**a**) 3D mapping of Her2^+^PD-L1^+^ (green) and Her2^+^PD-L1^−^ (red) cancer cells in a reconstructed mouse mammary tumor. Scale bar: 500 μm. (**b**) Relative quantification of Her2^+^PD-L1^+^ (green) and Her2^+^PD-L1^−^ (red) cancer cells out of total Her2^+^ cancer cells. (**c**) 3D cross-section image of **a** in Y-Z orthogonal plane. (**d**) Tomographic section image (at 900 μm Z-stack) showing distribution patterns of Her2^+^PD-L1^+^ (green) and Her2^+^PD-L1^−^ (red) cancer cells in the middle of the tumor. (**e**) Fluorescence intensity profile (Her2^+^PD-L1^+^ (green) and Her2^+^PD-L1^−^ (red)) along line indicated in **d**. (**f**) Heterogeneity of PD-L1 expression in different regions of the tumor. High resolution 2D images of Her2 and PD-L1 expression were obtained at the invasive margin, tumor core, and nearby tumor. Scale bar: 100 μm (left, right), 50 μm (middle). (**g**) Intensity profiling of Her2 and PD-L1 expression in tissue in **a**. Scattergram shows varying degrees of colocalized signal with a positive overall trend (Correlation coefficient = 0.77). (**h**) 3D spatial mapping of fluorescence intensity correlation of Her2 and PD-L1 biomarkers. Based on the product of differences from the mean (PDM) calibration bar (right insert), yellow and blue colors represent high and low correlations in the tumor, respectively. (**i**) Tomographic section image (at 900 μm Z-stack) of **h** showing the pattern of peripheral high (yellow) and central low (blue) intensity correlations of Her2 and PD-L1 in the tumor.

Analysis of 3D tumor imaging enabled by the T3 method revealed distinct relationships between PD-L1 expression and other components in the tumor microenvironment. To evaluate the colocalization of the tumor marker Her2 and PD-L1, we set a threshold for PD-L1 expression and mapped Her2^+^PD-L1^+^ and Her2^+^PD-L1^−^ cells (Fig. 3a). Overall, Her2^+^PD-L1^−^ cancer cells were more abundant than Her2^+^PD-L1^+^ cells (57% vs. 43%, Fig. 3b). Examining orthogonal Y-Z and X-Y virtual sections through the middle of the tumor revealed a consistent pattern where Her2^+^PD-L1^+^ cells were more abundant in the tumor periphery and Her2^+^PD-L1^−^ cells more concentrated in the tumor core (Fig.3c,d, Supplementary Fig. S5). Intensity profiles of PD-L1 immunoreactivity reinforced this pattern (Fig. 3e).

We then examined PD-L1 expression in distinct Her2-expressing tumor domains including the invasive margins, a nearby tumor focus, and the tumor core (Fig. 3f). PD-L1 expression by Her2^+^ cancer cells in the tumor periphery led to a brightly stained rim surrounding a core of mostly PD-L1^−^ Her2^+^ cancer cells. The small tumor focus displayed PD-L1 staining throughout. Insofar as the analysis was performed with completely stained macrosections (Supplementary Fig. S6), we were able to rule out antibody penetration artifact as a determinant of the pattern. Consistent with macrosection imaging, direct immunofluorescence analysis of 7 μm cryosections revealed a similar pattern where PD-L1 expression was limited to the tumor margin and absent from the core (Supplementary Fig. S7). As a complementary analysis, Her2 and PD-L1 staining intensity was plotted for each image pixel (Fig. 3g), which was converted to the product of the differences from the mean (PDM, Fig. 3h,i, Supplementary Fig. S8). The color-coded 3D PDM confirms a strong correlation of Her2 and PD-L1 expression at the margin and lack of co-expression in the core. Interestingly, despite the peripheral distribution, tumor cell location appeared to determine PD-L1 independently of proliferation per se. Although a previous 2D, IHC-based study reported a correlation between Ki-67 and PD-L1 expression in breast tumor cells^40^, our tumor-wide analysis showed divergent patterns of Her2^+^ cell expression of Ki-67 and PD-L1^+^ (Supplementary Fig. S9).

### 3D mapping of CD31^+^PD-L1^+^ and CD31^+^PD-L1^−^ endothelial cells

**Figure 4:**
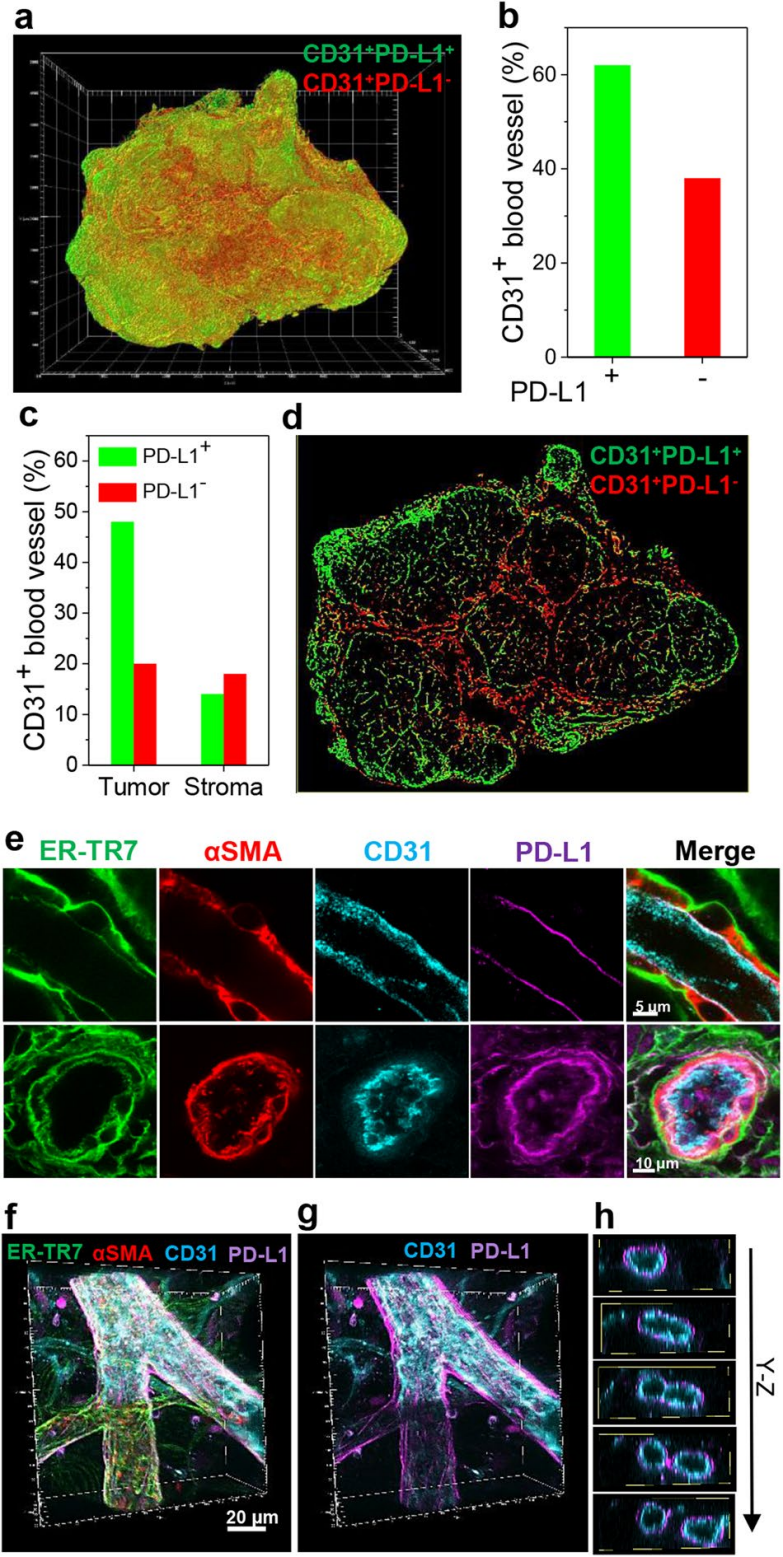
Spatial mapping and analysis of CD31 and PD-L1 expression. (**a**) 3D spatial mapping of CD31^+^PD-L1^+^ (green) and CD31^+^PD-L1^−^ (red) endothelial cells in a whole mouse mammary tumor. (**b**) Relative quantification of CD31^+^PD-L1^+^ (green) and CD31^+^PD-L1^−^ (red) endothelial cells out of total CD31^+^ endothelial cells in the whole tumor. (**c**) Relative quantification of CD31^+^PD-L1^+^ (green) and CD31^+^PD-L1^−^ (red) endothelial cells within the multi-lobed tumor mass and surrounding stroma in the whole tumor. (**d**) Tomographic section image (at 900 μm Z-stack) of **a** showing distribution patterns of CD31^+^PD-L1^+^ (green) and CD31^+^PD-L1^−^ (red) endothelial cells in the middle of the tumor. (**e**) PD-L1 expression in tumor blood vessels. High resolution longitudinal section (top) and cross-section (bottom) images of tumor blood vessels immunostained for ER-TR7 (green), αSMA (red), CD31 (cyan), and PD-L1 (magenta). Scale bar: 5 μm (top), 10 μm (bottom). (**f**) 3D rendering of a microvessel immunostained for ER-TR7 (green), αSMA (red), CD31 (cyan), and PD-L1 (magenta). Scale bar: 20 μm. (**g**) 3D rendering of PD-L1 and CD31 channel image in **f**. (**h**) Tomographic projection of **g** in Y-Z orthogonal plane. Note that PD-L1 expression appears to surround the CD31^+^ endothelium.

A significant part of the PD-L1 expression in the BALB-NeuT tumors was independent of Her2 immunoreactivity but associated with CD31^+^ staining, suggesting expression by blood vessels. 3D rendering of CD31^+^PD-L1^+^ and CD31^+^PD-L1^−^ blood vessels (Fig. 4a) facilitated determination of the fraction of PD-L1 positive vessels (Fig. 4b). While over half of the CD31^+^ blood vessels expressed PD-L1, tomographic imaging and quantitative analysis pointed to greater expression by the larger blood vessels in the tumor core (Fig. 4c,d, Supplementary Fig. S10), a reciprocal pattern to that of Her2^+^PD-L1^+^. To investigate the origin of vascular PD-L1 expression, we visualized PD-L1 along with vascular fibroblasts (TR-ER7), smooth muscle cells (αSMA), and endothelial cells (CD31) by staining separate BALB-NeuT mammary tumor macrosections (Fig. 4e). High resolution, longitudinal section and cross-section images placed PD-L1 expression between the endothelium and the inner layer of smooth muscle cells. 3D tomographic projection similarly localized PD-L1 expression to the sub-endothelial layer (Fig. 4f–h and Supplementary Video 5). Confirming this expression pattern, examining single macrosections cut from single lobed and multi-lobular masses (n = 6) revealed a similar distribution of PD-L1 expression to the periphery for Her2^+^ tumor cells and to the core for CD31^+^ endothelium (Supplementary Fig. S11).

### 3D mapping of infiltrating CD45^+^ immune cells with respect to PD-L1

**Figure 5:**
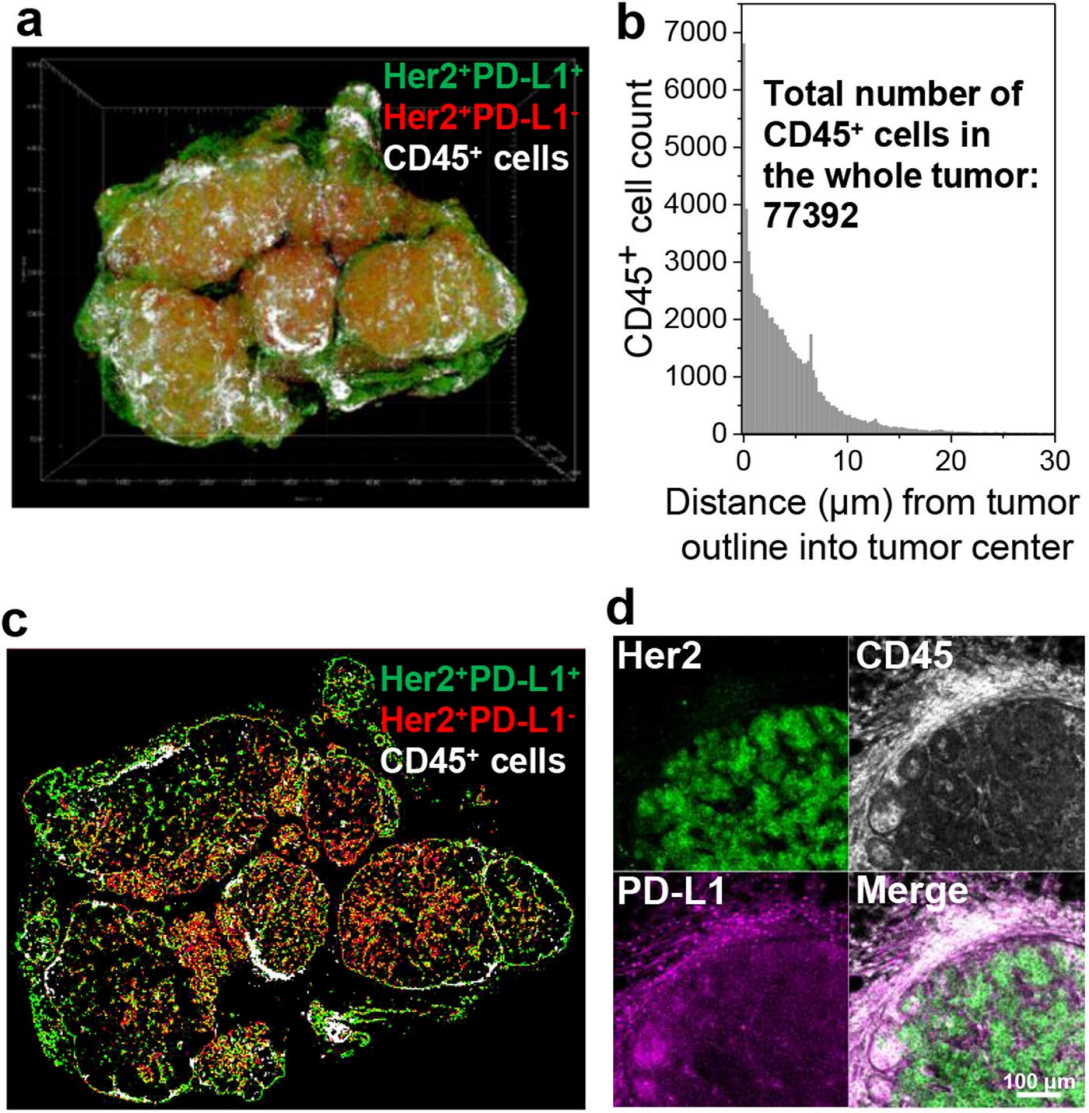
Spatial mapping of CD45^+^ immune cells. (**a**) 3D spatial mapping of CD45^+^ immune cells (white) with respect to Her2^+^PD-L1^+^ (green) and Her2^+^PD-L1^−^ (red) tumor domains. (**b**) 3D distance profile of CD45^+^ immune cells (total count in the whole tumor: 77,392) from the Her2^+^ tumor outline into the tumor center. (**c**) Tomographic section image (at 900 μm Z-stack) of **a** showing CD45^+^ immune cell (white) distribution in relation to Her2^+^PD-L1^+^ (green) and Her2^+^PD-L1^−^ (red) tumor domains. (**d**) High resolution 2D images for Her2, CD45, and PD-L1. High population of CD45^+^ immune cells located outside Her2^+^ and in PD-L1^+^ expression area.

In BALB-NeuT animals, mammary tumors arise and grow despite the intact immune system of the host. One mechanism of immunosuppression mediated by tumor cell expression of PD-L1 may be via inducing exhaustion in infiltrating cytotoxic T cells expressing PD-1 receptors^41^. To examine the distribution of immune infiltrate in NeuT tumors, we mapped CD45^+^ cells in relation to Her2^+^PD-L1^+^ and Her2^+^PD-L1^−^ tumor cells (Fig. 5 and Supplementary Fig. S12). Dense clusters of CD45^+^ immune cells were located at the tumor edge with far fewer immune cells infiltrating the tumor, leaving much of the volume non-inflamed (Fig. 5a–c). Strikingly, at high magnification, clusters of CD45^+^ immune cells were visualized within PD-L1^+^ normal tissue just outside the border of Her2 immunoreactivity (Fig. 5d).

## Discussion

Here, we report transparent tissue tomography (T3) and its application to mapping patterns of biomarker expression in tumors and the microenvironment over the scale of cells to whole tumors. Immune detection of biomarkers in tumor tissue by immunohistochemistry (IHC) is a fundamental tool not only for preclinical research but also clinical diagnostics. The standard workflow of formaldehyde fixation, embedding in paraffin, thin sectioning, deparaffinization, epitope retrieval, and probing with primary antibodies and secondary enzyme conjugates followed by slide scanning, provides cell-resolution immunolocalization in one plane and of one or two antigens at a time. Although IHC can be adapted to three dimensional analysis^42^, it is impractical to cut, process and image serial sections over millimeter depths. Whole mount methods bypassing the need to cut sections have long been applied to immunolocalization in tumors^43,44^, but these approaches are limited to small and thin tissue samples. Challenges have included rendering the tumor tissue sufficiently transparent while maintaining structural integrity and protein immunogenicity, obtaining satisfactory penetration of antibodies and conjugates, and then performing multiplexed detection with high sensitivity and specificity at cellular resolution. To overcome these limitations and enable imaging of whole tumors, we examined the feasibility of a tomographic strategy where thick slices are cut from the tissue, processed and imaged, and then the digital images registered and knit together to reconstruct the tumor. We found that slicing tumors into 400 μm macrosections enabled simple and rapid immunofluorescence staining, optical clearing, and confocal microscopic imaging.

Immunolabeling remains a considerable barrier to 3D tissue analysis^23,45^. Antibody staining for intact organs such as the mouse brain can require several days^46,47^. By contrast, 400 μm macrosections were readily stained overnight with multiple fluorescent primary antibodies. In turn, macrosectioning also facilitated tissue clearing. Optical clearing of a whole mouse brain by equilibration with concentrated D-fructose (115% w/v) as in the SeeDB protocol may require a week of incubation^28^. Although diffusion rates of a clearing agent into different tissue structures may be distinct^48^, incubating tumor macrosections in increasing concentrations of D-fructose was sufficient to complete equilibration to 80% w/v D-fructose within 3 h. Another advantage was that once optically cleared, tumor macrosections could be fully scanned using a standard 10X air objective (e.g. 10X/0.4 NA, 2.2 mm working distance) and conventional confocal microscope. The resulting voxel dimensions, though undersampled with respect to the Nyquist criterion, were satisfactory for rapid surveys and mapping down to cellular resolution. Subsequently, areas of interest could be examined at higher resolution (e.g. 40X/1.25 NA, 0.24 mm working distance), though at cost to scan and data analysis time. Finally, to efficiently reconstruct and analyze whole tumors, we adapted open-source Fiji plugins to perform as semi-automated macros. The tomographic reconstruction displayed sufficient consistency that we could develop and apply open-source macros to segment tumors and quantify multiple parameters including different cell types, patterns of biomarker expression, and distances between cells.

Using an optimized T3 protocol, we were able to probe the expression of PD-L1 throughout tumors and the microenvironment, capturing heterogeneity at multiple scales. In the BALB-NeuT tumors, we observed higher PD-L1 immunoreactivity in the periphery, apparently expressed not only by Her2^+^ cancer cells but also components of the stroma. Regions of clustered CD45^+^ cells, with size and shape consistent with tumor-infiltrating lymphocytes (TILs), were localized at the tumor edge, consistent with prior observations^49,50^. Though the immune cells appear as if they may be reacting to the tumor, they are likely restrained by PD-L1 signaling, raising the question whether a key impact of anti-PD-L1 agents is to restore activity of infiltrating T cells poised at the tumor margin.

Distinct from the pattern at the tumor margins and periphery, T3 analysis revealed far fewer tumor cells expressing PD-L1 in the tumor core. However, blood vessels in the tumor core displayed strong PD-L1 immunostaining, apparently localized to the membrane of endothelial cells in contact with the basal lamina but absent from the luminal surface. PD-L1 expression by endothelial cells has been described^51^ and proposed to limit immune cell extravasation^52^ with potential impacts on activity or adverse effects of anti-PD-L1 antibody therapy^53^. Notably, in the tumor core, where blood vessels display the most striking expression of PD-L1, we observed few infiltrating CD45^+^ cells, consistent with a barrier to extravasation.

In summary, we report transparent tissue tomography (T3) as a new tool for 3D spatial imaging and analysis of the tumor microenvironment. Slicing tumors into 400 μm macrosections enables simple and rapid immunofluorescence staining, optical clearing, and confocal microscope imaging. Registering and fusing macrosection images yields high resolution 3D maps of multiple biomarkers throughout a tumor which can be evaluated by automated image analysis. By assessing multiple tumor parameters simultaneously, T3 provides a unique window into the heterogeneity of the tumor immune microenvironment. We anticipate that T3 can be applied broadly to facilitate preclinical studies of tumor biology and therapy. In particular, spatial, multiparameter T3 analysis may serve as a tool to improve diagnostic, prognostic and predictive testing of patient biopsies as part of evaluation for immune checkpoint blockade therapy.

## Methods

### Mouse tumor models

Transgenic BALB/c males carrying the mutated rat Her2/neu oncogene driven by the MMTV promoter were bred with wild type females^39^. Genotypes of offspring were determined by PCR of tail snips. BALB-NeuT female mice developed spontaneous mammary carcinoma in each mammary gland between 5 and 33 weeks of age. All mice were maintained under specific pathogen-free conditions in accordance with the animal experimental guidelines set by the Institutional Animal Care and Use Committee. The study has been approved by the Institutional Animal Care and Use Committee of the University of Chicago and all experiments conformed to the relevant regulatory standards.

### Antibodies

Anti-rat Her2 antibody was isolated from culture supernatant from hybridoma 7.16.4 (ATCC) at the Frank W. Fitch Monoclonal Antibody Facility at the University of Chicago. Primary antibodies diluted in phosphate buffered saline pH 8.0 (PBS, Corning) were conjugated to fluorescent dyes as shown in Supplementary Table S1. Reactions were incubated with gentle agitation at 4 °C overnight. Unreacted dye was removed by dialysis (MWCO 10 K) against PBS pH 7.4 at 4 °C for 3 days. We also purchased Cy3-conjugated anti-αSMA antibody (1A4, Sigma Aldrich). Fluorescent antibody solutions were stored at 4 °C.

### Tumor macrosectioning

Tumors were harvested, washed in cold PBS, fixed with 2% paraformaldehyde in PBS for 10 min at room temperature, and washed in PBS. Then, tumors were cast in 2% agarose gel (dissolved in distilled water, LE Quick Dissolve Agarose, GeneMate) in 12 well plates. The gel plugs containing tumors were marked for orientation and mounted on a vibrating microtome (VT1200S, Leica) equipped with a buffer tray. Sections were collected in order in cold PBS, and fixed with 2% paraformaldehyde in PBS for 5 min at room temperature.

### Immunofluorescence staining

Tumor macrosections were stained with antibody cocktails in staining buffer (SB, PBS pH 7.4 with 10 mg/ml BSA and 0.3% Triton X-100). For Fig. 2 and Supplementary Fig. S4, 40 μl DyLight488-anti-Her2, 20 μl DyLight550-anti-CD45, 20 μl DyLight594-anti-Ki-67, 5 μl DyLight-633-anti-CD31, and 200 μl DyLight680-anti-PD-L1 were combined in 1 ml SB. For Fig. 4e–h, 10 μl DyLight488-anti-reticular fibroblast, 2.5 μl Cy3-anti-αSMA, 2.5 μl DyLight633-anti-CD31, and 100 μl DyLight680-anti-PD-L1 were combined in 0.5 ml SB. To test antibody penetration, the macrosections shown in Supplementary Fig. S2a were incubated with 40 μl DyLight633-anti-Her2 antibody in 1 ml SB for 1, 4, 11, and 21 h at 4 °C. All the other macrosections shown in the remaining Figures and Supplementary Figures were incubated with the antibody cocktails for 18 h at 4 °C. After incubation, macrosections were washed three times for 10 min in PBS pH 7.4 at 4 °C, fixed with 2% paraformaldehyde in PBS for 10 min at room temperature, and washed in PBS pH 7.4.

### Cryosectioning and immunofluorescence staining

Tumors were embedded in optimal cutting temperature (O.C.T., Tissue-Tek) compound, frozen at -80 °C and sectioned at 7 µm thickness in a cryostat at -20 °C. Sections were transferred to microscope slides and dried at room temperature. After rehydrating in PBS, sections were stained with the DyLight488-anti-Her2 and DyLight680-anti-PD-L1, washed and imaged by confocal microscopy.

### Optical clearing of tumor macrosections

To prepare D-fructose solutions for optical clearing, 0, 20, 40, 60, 80, and 100% (w/v) solutions of D-fructose were prepared in 10 mM phosphate buffer pH 7.8 to a final volume of 10 ml. SeeDB solution (~115% D-fructose) was prepared by dissolving 20.25 g D-fructose in 5 ml phosphate buffer. 30 μl of α-thioglycerol (Sigma Aldrich) was added to each D-fructose solution. 0.5 mm and 1.0 mm macrosections were incubated sequentially in 10 ml of each D-fructose solution for 1 h at 25 °C with gentle agitation. After each incubation, transparency was evaluated by measuring transmittance (%) over the range from 350 nm to 1000 nm using a UV/Vis spectrophotometer (Agilent 8453, Agilent) as described^48^. Immunostained tumor macrosections (at 400 μm thickness) were incubated sequentially in 10 ml of 20, 50, and 80% D-fructose solutions for each 1 h at 25 °C with gentle agitation.

### Confocal microscopic imaging

We used a Leica SP5 AOBS II tandem scanner spectral confocal microscope, Leica HCX PL APO 10X/0.4 NA dry objective (2.2 mm working distance) or Leica HC PL APO 40X/1.25 NA oil objective (0.24 mm working distance), and a SuperZ galvometric scanning stage to image immunostained macrosections mounted between coverslips in 80 w/v% D-fructose solutions. 3D scanning was performed in a defined X/Y/Z (1.78/1.78/6.25 μm) volume with 8 frame averaging and bidirectional scanning using 488 nm (Argon) excitation and 495–528 nm emission filter for DyLight488, 514 nm (Argon) and 558–575 nm filters for DyLight550 and Cy3, 561 nm (DPSS) and 585–614 nm filter for DyLight594, 633 nm (HeNe) and 637–655 nm filter for DyLight633, and 633 nm (HeNe) and 713–752 nm filter for DyLight680.

### Image processing

We developed macros for automated 3D reconstruction of whole tumor images using open-source plugins in Fiji (http://fiji.sc/Fiji). Using the grid/collection stitching plugin^54^, we built macro *LIFtile-restitcher* to align and stitch 3D mosaics for multi-channel “hyperstack” images (0–999 image tiles). To compensate for depth-related intensity losses, the mean intensity value (of Otsu-thresholded areas; maximum gain limited to 3X) of each optical slice in the macrosection images was normalized to the mean value of the entire macrosection for each channel using the macro *HPRstackConstantMean*, facilitating thresholding and segmentation as used in cell enumeration. Using the MultiStackReg plugin (http://bradbusse.net/sciencedownloads.html), we designed macro *composite big aligner* that enabled automated alignment and registration of the stitched macrosection images. To remove gaps between macrosections in the 3D reconstructed tumor image, the macro *closeZvoids* merged the top and bottom of adjoining macrosections by summing signal from two adjoining optical slices. Next, we used macro *hyprBKGDfix* to clear background outside the stained tissue to facilitate 3D volume visualization. Finally, the 3D images were deconvolved using Huygens Pro software v. 4.3 (Scientific Volume Imaging), to correct for refractive index mismatch, and to enhance spatial signals and cell morphology discrimination. We constructed 3D tomographic visualizations and movies of the tumor images using Imaris software v. 8.3.1 (Bitplane).

### Data analysis

For hyperstack segmentation of Her2^+^ and PD-L1^+^ cells, we determined the cutoff threshold for the Her2 and PD-L1 channel images, and converted into binary (8 bit) images. We developed the macro *vessel extractor* for automated segmentation of CD31^+^ blood vessels from the CD31 channel image. For hyperstack segmentation of CD45^+^ cells from the images, we built macro *wekaMacro* using the trainable Weka segmentation plugin^55^. The classifier was trained to extract the cells only showing fluorescence signals on the membrane. To study the intensity correlation of Her2 and PD-L1, we used the Intensity Correlation Analysis plugin^56^. Also, to extract tumor outlines, we created macro *HER2outlinerMacro*. For measuring distance of CD45^+^ immune cells from the tumor edge, we applied the 3D distance map plugin to the tumor outlines^57^. All other image analysis was conducted with basic analyze functions in Fiji software.

### Code availability

Fiji macros (Supplementary Table S2 and Fiji macro script 1–8) can be downloaded at the Electronic Supplementary Material.

## Electronic supplementary material

Supplementary Information

Supplementary Video 1

Supplementary Video 2

Supplementary Video 3

Supplementary Video 4

Supplementary Video 5
