# Supplementary material for: Multiplex three-dimensional optical mapping of tumor immune microenvironment
Source: Sci Rep. 2017 Dec 5;7:17031. doi: 10.1038/s41598-017-16987-x (PMC5717053; doi:10.1038/s41598-017-16987-x)
Supplement: Supplementary file 1 — Supplementary Information [file 41598_2017_16987_MOESM1_ESM.pdf]

Supplementary Information for

Multiplex three-dimensional optical mapping of tumor immune microenvironment

Steve Seung-Young Lee, Vytautas P. Bindokas, Stephen J. Kron

File includes:

Supplementary figures S1-12

Supplementary tables S1, S2

Fiji macro scripts 1-8

Supplementary videos 1-5

Supplementary Figure S1. Optimizing optical clearing of tumor macrosections. (**a**) Optical transparency spectrum of 500 μm and 1000 μm thickness tumor macrosections following sequential incubation in D-fructose solutions at indicated concentrations (%, w/v). The transparency was determined by measuring light transmittance (%) over the range from 350 nm to 1000 nm. (**b**) Qualitative effect of optical clearing on tissue transparency, showing 1000 μm macrosection before (top) and after (bottom) equilibration with 80 % D-fructose solution.

Supplementary Figure S2. Optimizing antibody penetration and multiplex immunostaining for 400 μm macrosections. (**a**) Optical cross-section (Z) scanning of optically cleared macrosections (at 400 µm thickness) after incubation with or without anti-Her2 antibody for different times (1, 4, 11, and 21 h). (**b**) Optical cross-section (Z) scanning of an optically cleared macrosection (at 400 µm thickness) after immunostaining for Her2 (green), CD45 (yellow), Ki-67 (red), CD31 (cyan), and PD-L1 (magenta). X-Z projections in top row, X-Y views in bottom row.

Supplementary Figure S3. Optimizing confocal microscopy conditions for 400 μm macrosections. (**a**) 3D rendering of one macrolayer. Bottom image shows a side view of the 400 μm thick volume. (**b**) 3D rendering of a block in the red dotted square region in **a**. The dimensions of the block are 580 x 580 x 400 µm. (**c**) Virtual sections of **b** at Z positions 0 μm (top), 200 μm (middle), and 400 µm (bottom), demonstrating consistent staining throughout the macrosection.

Supplementary Figure S4. 3D spatial visualization of multiple tumor microenvironment components and biomarkers in a whole BALB/c NeuT tumor. (**a**) 3D rendering of tumor image reconstructed from six 400 μm macrosections after fluorescent immunostaining for Her2 (green), CD45 (yellow), Ki-67 (red), CD31 (cyan), and PD-L1 (magenta). Scale bar: 500 μm. Left top insert is tumor tissue prior to macrosectioning. (**b**) Side view of tumor image reconstructed from six macrosection images. White indicates superimposition of multiple colors. Scale bar: 500 μm. (**c**) Tomographic visualization of reconstructed tumor image with multiple orthogonal views (X-Y, X-Z, Y-Z planes). Surface distortions of macrosection faces can result in small gaps. These defects can be corrected with image warping-based alignment methods, but they typically produce only minor distortions as in the reassembled tumor here. (**d**) Separated 3D projections for each cellular marker or biomarker in the tumor.

Supplementary Figure S5. Spatial mapping and analysis of Her2 and PD-L1 expression. (**a**) 3D spatial mapping of $Her2^+PD\text{-}L1^+$ (green) and $Her2^+PD\text{-}L1^-$ (red) cancer cells in the BALB/c NeuT tumor shown in Supplementary Figure S4. Scale bar: 500 μm. (**b**) Volume percentages (%) of $Her2^+PD\text{-}L1^+$ (green) versus $Her2^+PD\text{-}L1^-$ (red) tumor cells in **a**. (**c**) 3D cross-section image of **a** in Y-Z orthogonal plane. (**d**) Tomographic section image (at 820 μm in the Z-stack) of **a** showing distribution patterns of $Her2^+PD\text{-}L1^+$ (green) and $Her2^+PD\text{-}L1^-$ (red) cells in the middle of the tumor. (**e**) Fluorescence intensity profile ($Her2^+PD\text{-}L1^+$ (green) and $Her2^+PD\text{-}L1^-$ (red)) corresponding to white line in **d**. Note the preferential distribution of $Her2^+PD\text{-}L1^+$ to tumor edges versus $Her2^+PD\text{-}L1^-$ to the center.

Supplementary Figure S6. Parallel patterning of PD-L1 expression throughout the macrosections. (**a** and **b**) Optical X-Y section images of top and middle layers of the

macrosections (shown in the Main Figures (**a**) and the Supplementary Figure (**b**)) showing the parallel patterns of PD-L1 expression. These data indicate homogenous antibody penetration and immunostaining throughout the macrosections.

Supplementary Figure S7. Immunolocalization of Her2 and PD-L1 expression in frozen tumor section. Images of Her2 and PD-L1 expression at tumor margin and core in cryosectioned BALB/c Neu T tumor slide (at 7 μm thickness). Insert in the top row right is zoomed-in image at tumor edge and stroma. White indicates imposition of Her2 (green) and PD-L1 (magenta) signals. Scale bar: 20 μm.

Supplementary Figure S8. Intensity correlation analysis of Her2 and PD-L1 expression. (**a**) Cytofluorogram of Her2 and PD-L1 expression in the BALB/c NeuT tumor shown in Supplementary Figure S4 (Correlation coefficient=0.45). (**b**) 3D spatial mapping of intensity correlation of Her2 and PD-L1 biomarkers, where yellow and blue hues represent high and low correlations in the tumor, respectively. (**c**) Tomographic section image at 820 μm in the Z-stack of **b** showing peripheral high (yellow) and central low (blue) correlation of Her2 and PD-L1 expression across the tumor.

Supplementary Figure S9. Distinct patterns of expression of Ki-67 and PD-L1 in tumor cells. Tomographic section image at 900 μm in the Z-stack of Figure 2a showing overall lack of correlation between proliferating Ki-67-positive cells (red) and $Her2^{+}PD\text{-}L1^{+}$ tumor cells (green). $Her2^{-}$ stromal cells also express Ki-67.

Supplementary Figure S10. 3D spatial mapping of CD31 and PD-L1 expression. (**a**) 3D rendering of $CD31^+PD\text{-}L1^+$ (green) and $CD31^+PD\text{-}L1^-$ (red) endothelial cells in the BALB/c NeuT tumor shown in Supplementary Figure S4. (**b**) Volume percentages (%) of $CD31^+PD\text{-}L1^+$ (green) and $CD31^+PD\text{-}L1^-$ (red) endothelial cells out of total $CD31^+$ endothelial cells in **a**. (**c**) Tomographic section image at 820 μm in the Z-stack of **a** showing distribution patterns of $CD31^+PD\text{-}L1^+$ (green) and $CD31^+PD\text{-}L1^-$ (red) endothelial cells at the middle of the tumor. (**d**) Volume percentages (%) of $CD31^+PD\text{-}L1^+$ (green) and $CD31^+PD\text{-}L1^-$ (red) endothelial cells within tumor parenchyma and surrounding stroma shown in **a**.

Supplementary Figure S11. Spatial mapping and analysis of PD-L1 expression in macrosections from multiple tumors (n=6). (**a**) 3D rendering of BALB/c NeuT tumor macrosections (400 μm) each immunostained for Her2 (green), CD31 (cyan), and PD-L1 (magenta). Scale bar: 500 μm. 3D mapping of $Her2^+PD\text{-}L1^+$ (green) and $Her2^+PD\text{-}L1^-$ (red) cancer cells in the macrosections. 3D spatial mapping of $CD31^+PD\text{-}L1^+$ (green) and $CD31^+PD\text{-}L1^-$ (red) blood vessels. (**b**) Fluorescence intensity profiles of $Her2^+PD\text{-}L1^+$ (green) and $Her2^+PD\text{-}L1^-$ (red) along lines as indicated in **a**. (**c**) Relative quantification of $CD31^+PD\text{-}L1^+$ (green) and $CD31^+PD\text{-}L1^-$ (red) endothelial cells out of total endothelial cells.

Supplementary Figure S12. Spatial mapping of $CD45^+$ cells. (**a**) Location of $CD45^+$ immune cells (white) in relation to $Her2^+PD\text{-}L1^+$ (green) and $Her2^+PD\text{-}L1^-$ (red) tumor region borders for the tumor shown in Supplementary Figure S4. (**b**) Tomographic section image at 820 μm in the Z-stack of **a** showing $CD45^+$ immune cells (white) in relation to outlined regions of predominantly $Her2^+PD\text{-}L1^+$ (green) and $Her2^+PD\text{-}L1^-$ (red) tumor cells. (**c**) 3D distance profile

of total $CD45^+$ immune cells (count: 18,547) with respect to the outer edge of $Her2^+$ tumor cells. Note that most $CD45^+$ immune cells are located within the PD-L1-expressing outer layer of the tumor.

Supplementary Table S1. Fluorescent antibody conjugation

Supplementary Table S2. Fiji macros for 3D image processing and analysis

Fiji macro script 1. LIFtile-restitcher

Fiji macro script 2. HPRstack2ConstantMean

Fiji macro script 3. composite big aligner

Fiji macro script 4. closeZvoids

Fiji macro script 5. hyprBKGDfix

Fiji macro script 6. wekaMacro

Fiji macro script 7. vessel extractor

Fiji macro script 8. HER2outlinerMacro

Supplementary Video Legends

Supplementary video 1. 3D rendering of multiple tumor microenvironment components and biomarkers in a BALB/c NeuT tumor. Video shows spatial distributions of Her2 (green), CD45 (yellow), Ki-67 (red), CD31 (cyan), and PD-L1 (magenta) in the tumor.

Supplementary video 2. 3D rendering of an image block within a BALB/c NeuT tumor. Video shows detailed morphological features and biomarker distributions in the tumor microenvironment. Her2 (green), CD45 (yellow), Ki-67 (red), CD31 (cyan), and PD-L1 (magenta).

Supplementary video 3. Tomographic visualization of reconstructed BALB/c NeuT tumor image with multiple orthogonal planes. Her2 (green), CD45 (yellow), Ki-67 (red), CD31 (cyan), and PD-L1 (magenta).

Supplementary video 4. Tomographic section image of BALB/c NeuT tumor (at z=900 μm). Her2 (green), CD45 (yellow), Ki-67 (red), CD31 (cyan), and PD-L1 (magenta).

Supplementary video 5. High resolution 3D rendering of PD-L1 expression in blood vessels in BALB/c NeuT tumor. ER-TR7 (green), αSMA (red), CD31 (cyan), and PD-L1 (magenta).

Supplementary Figure S1

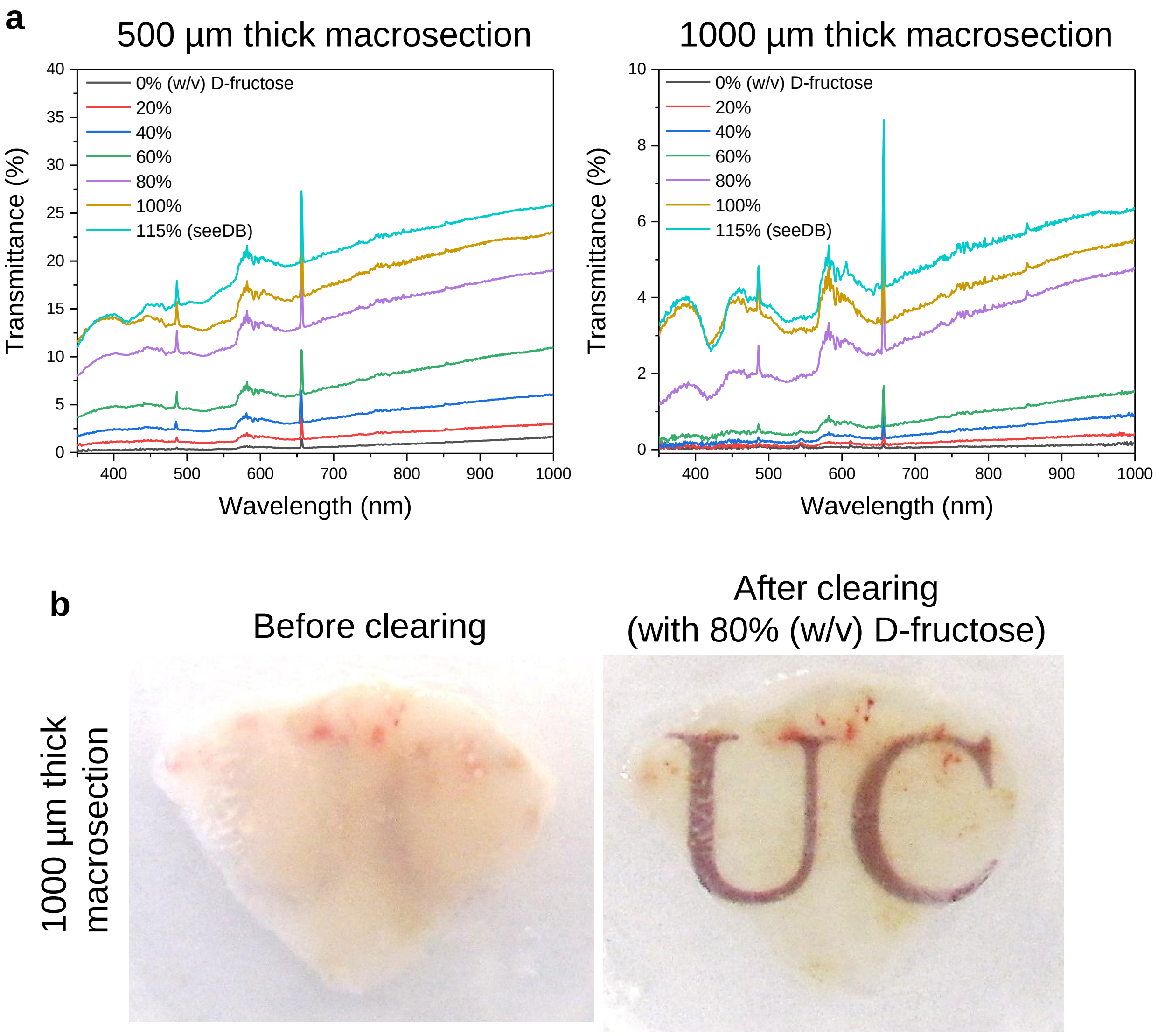

Supplementary Figure S2

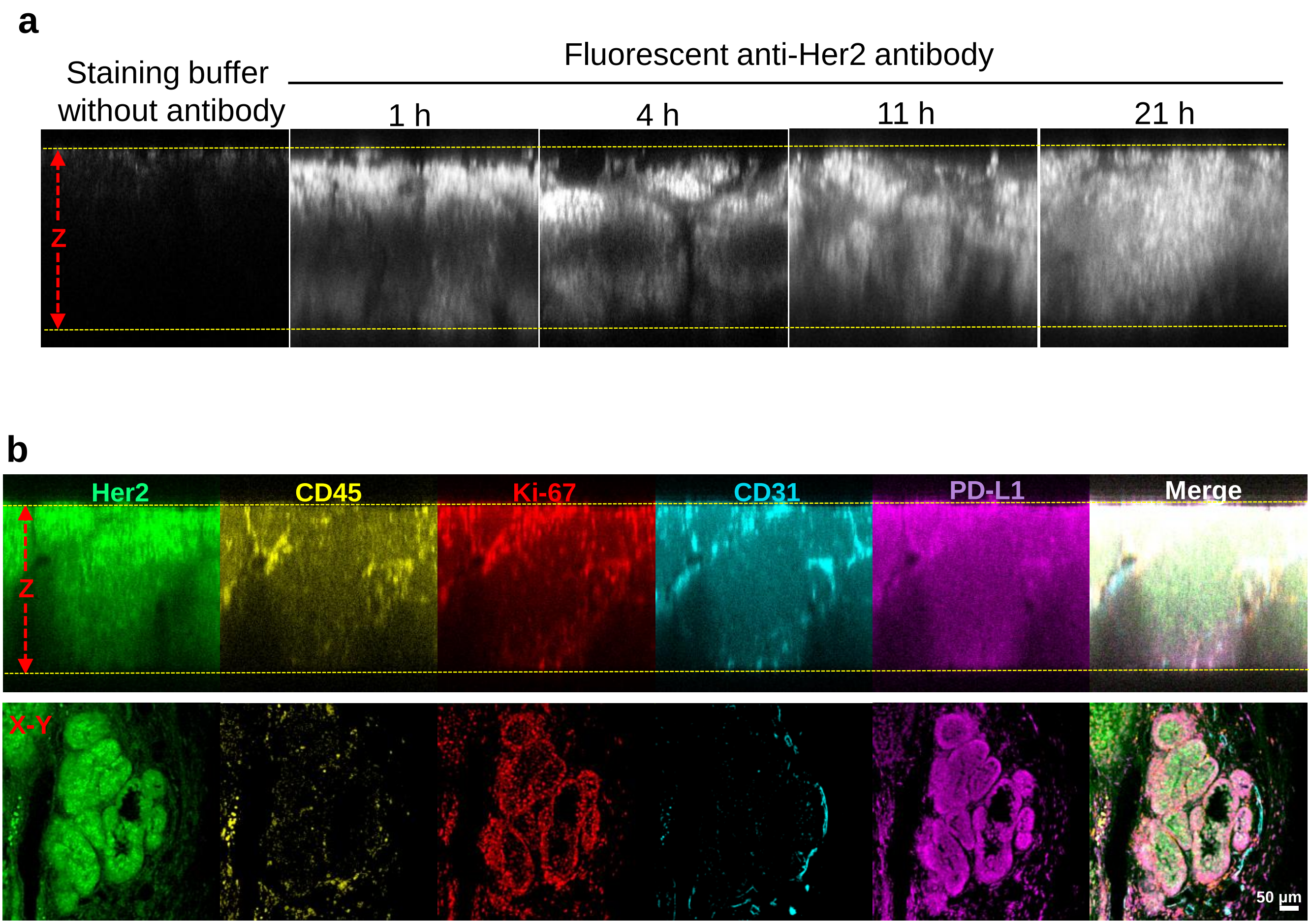

Supplementary Figure S3

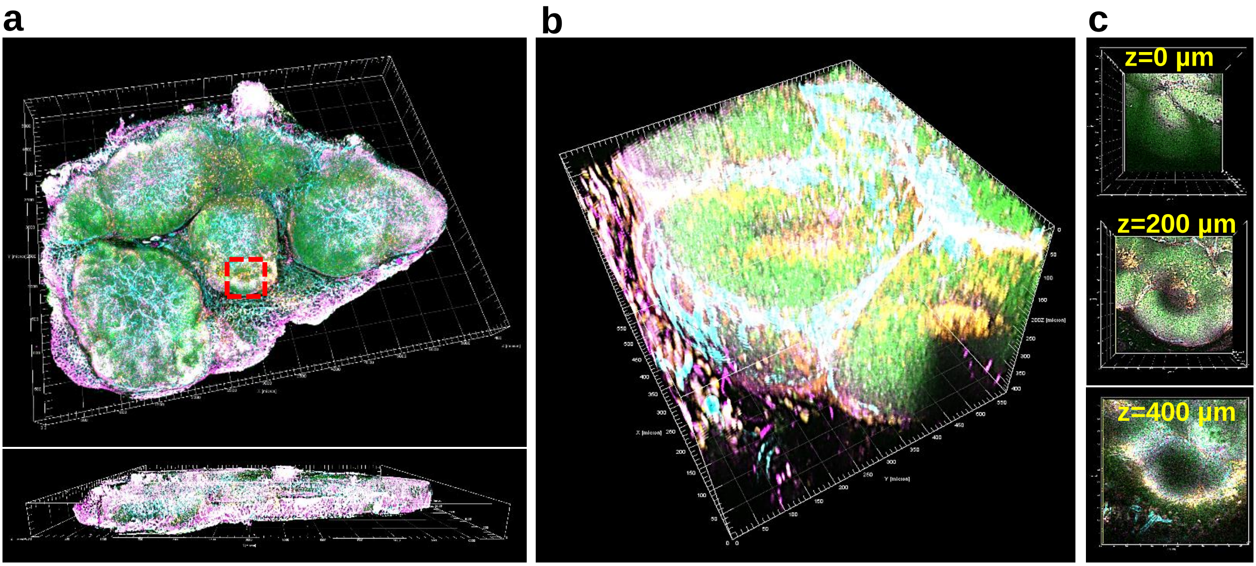

Supplementary Figure S4

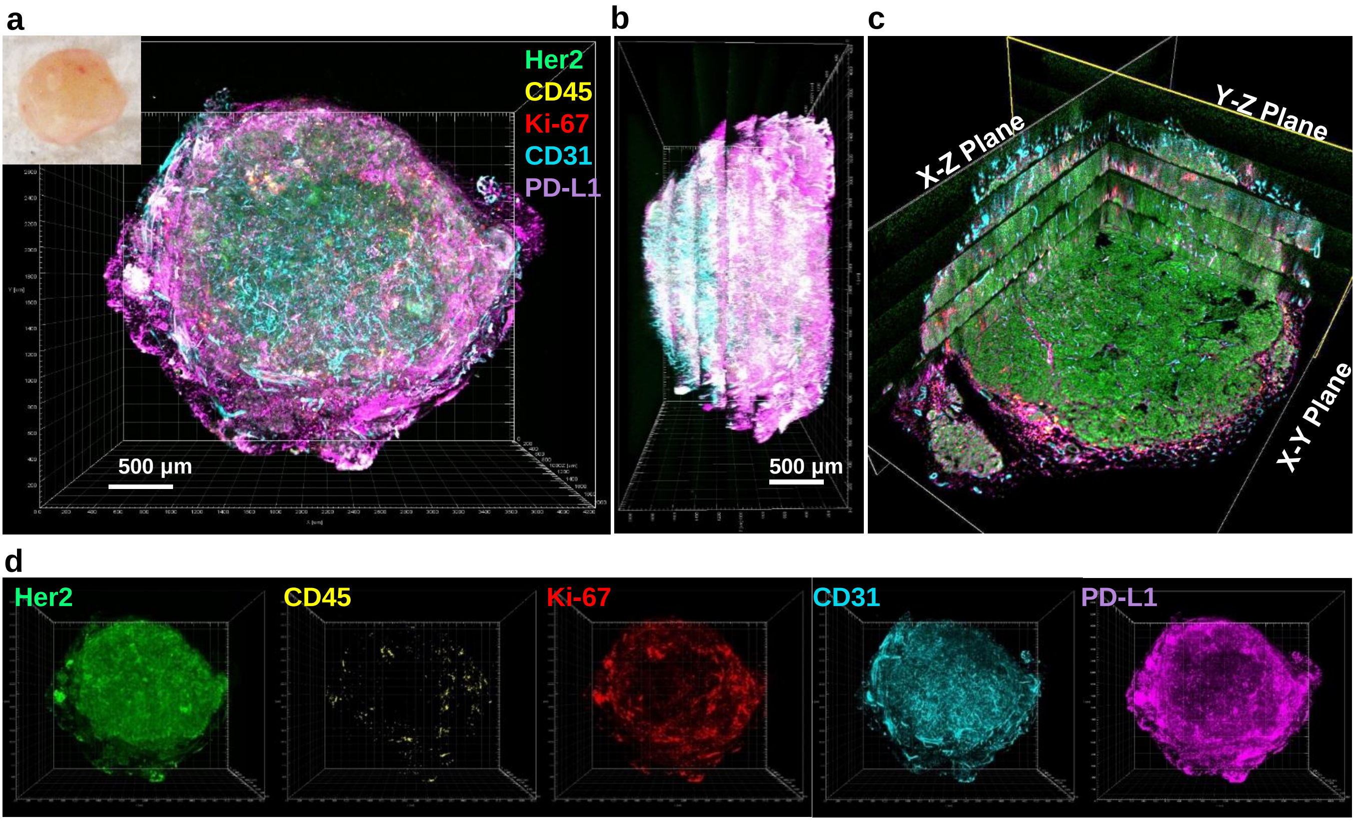

Supplementary Figure S5

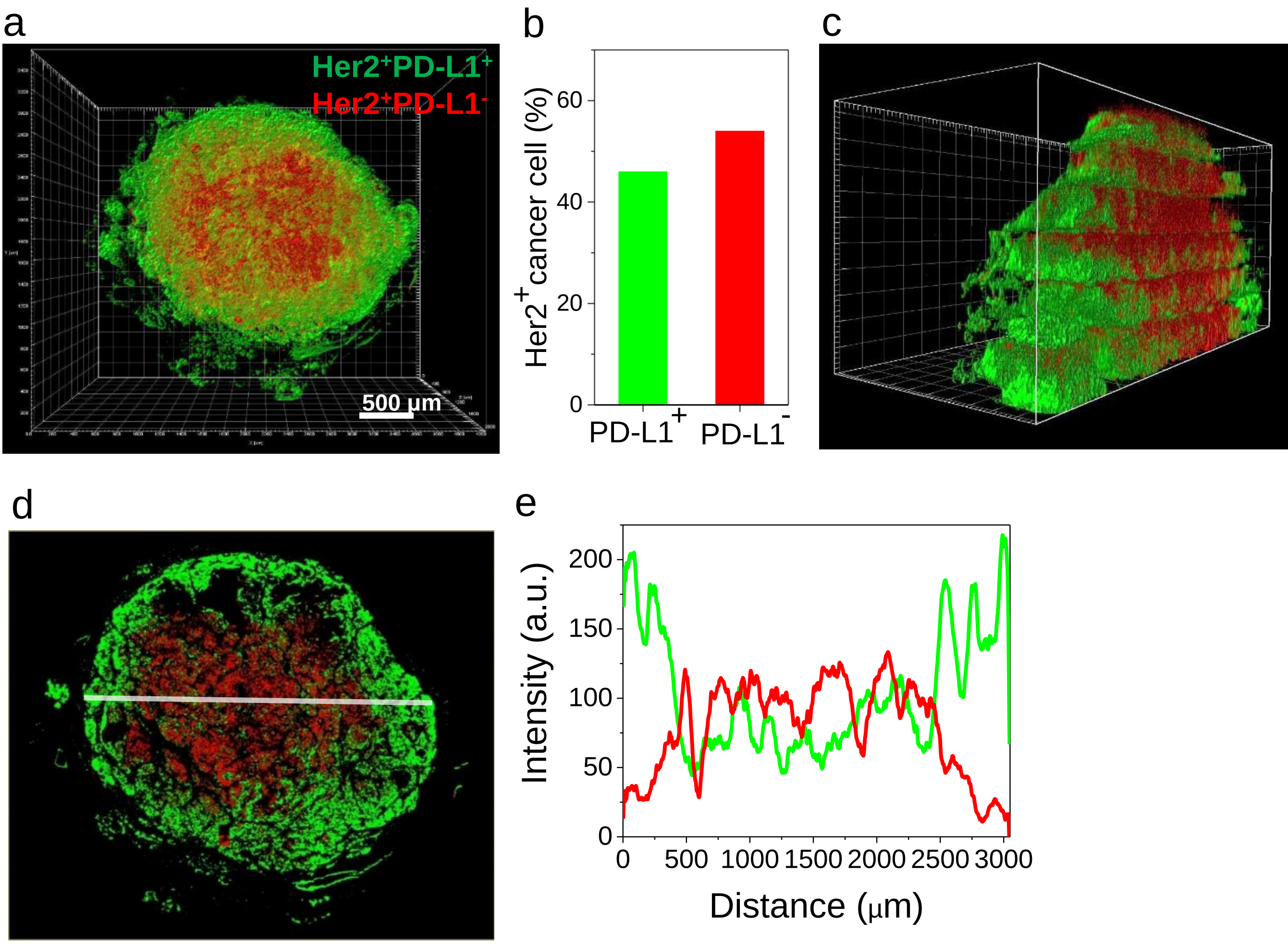

Supplementary Figure S6

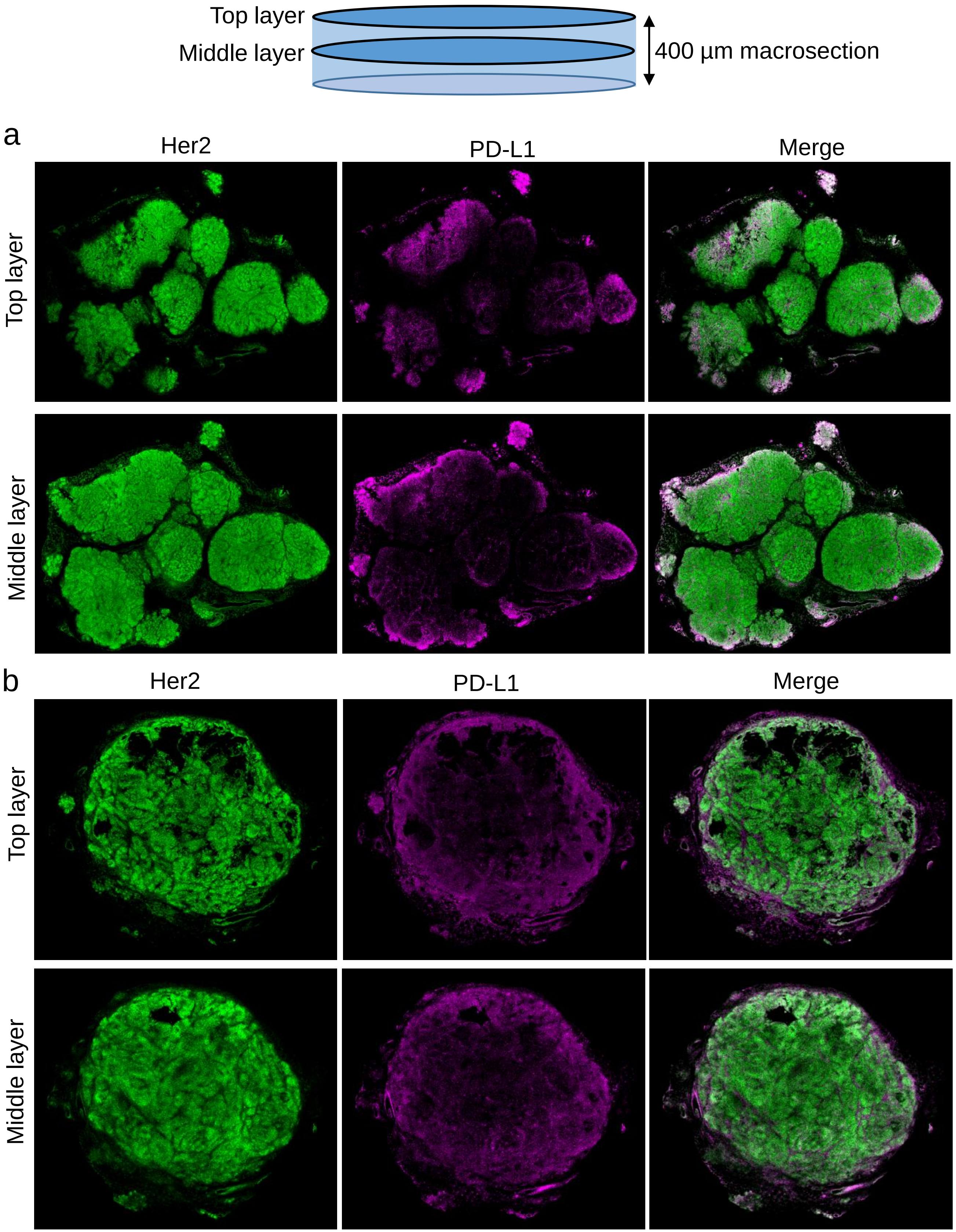

Supplementary Figure S7

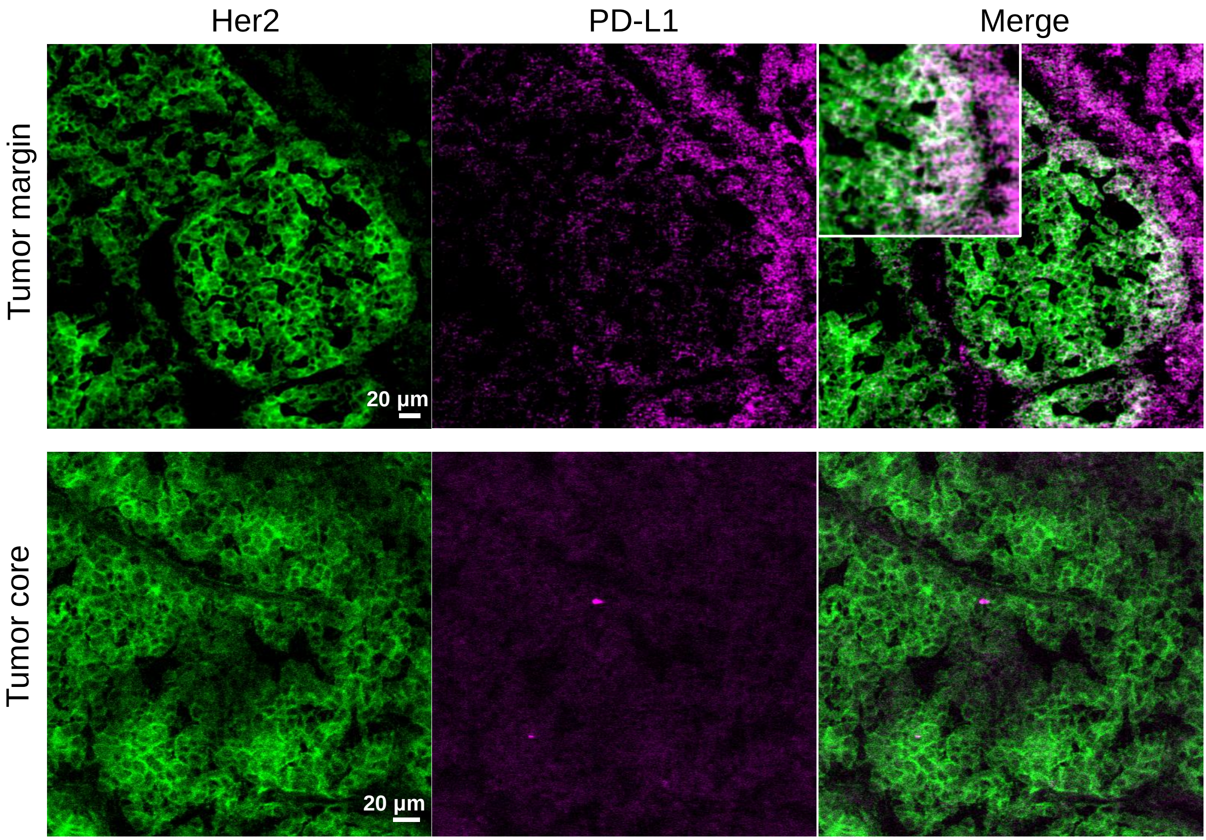

Supplementary Figure S8

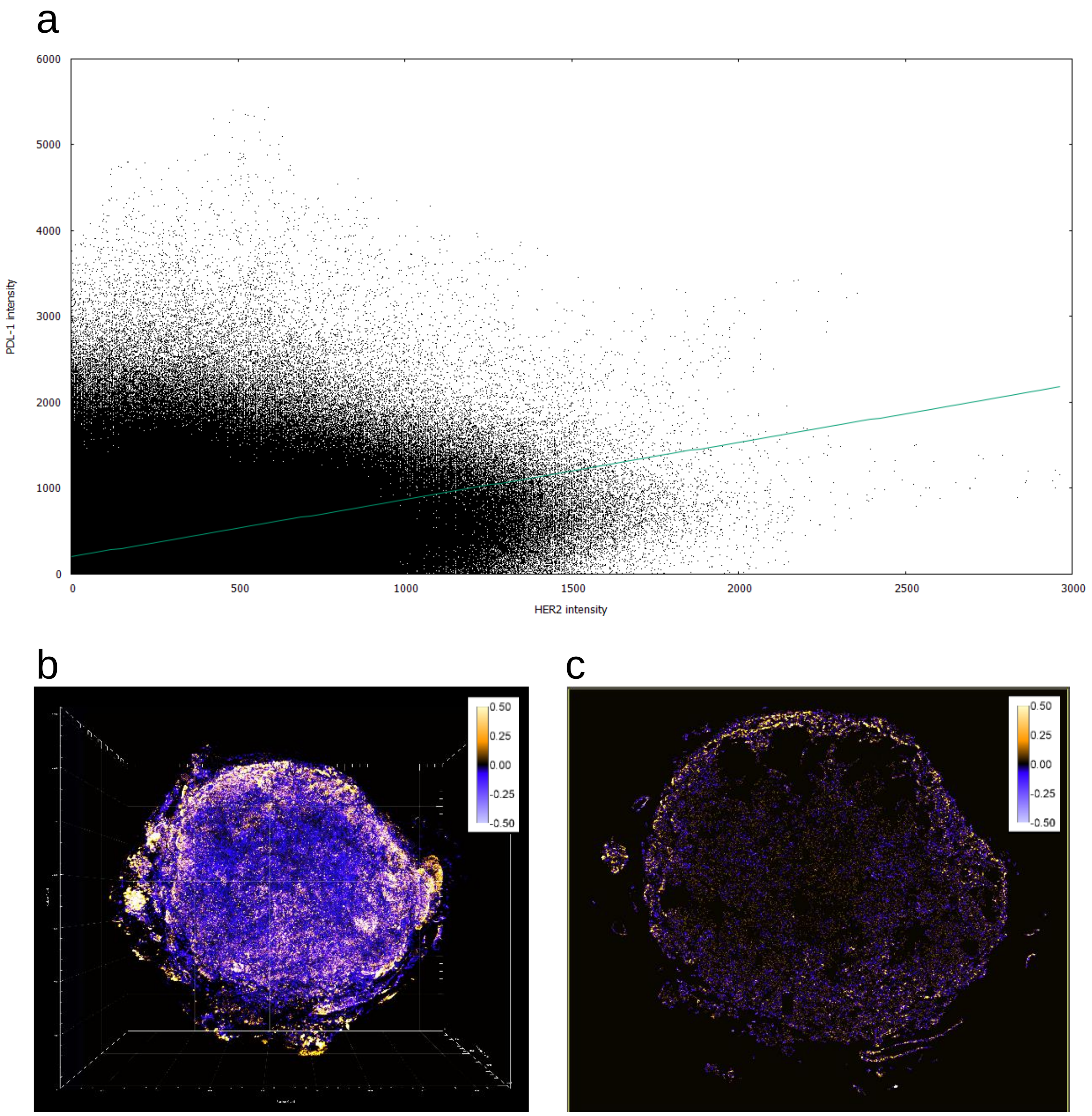

Supplementary Figure S9

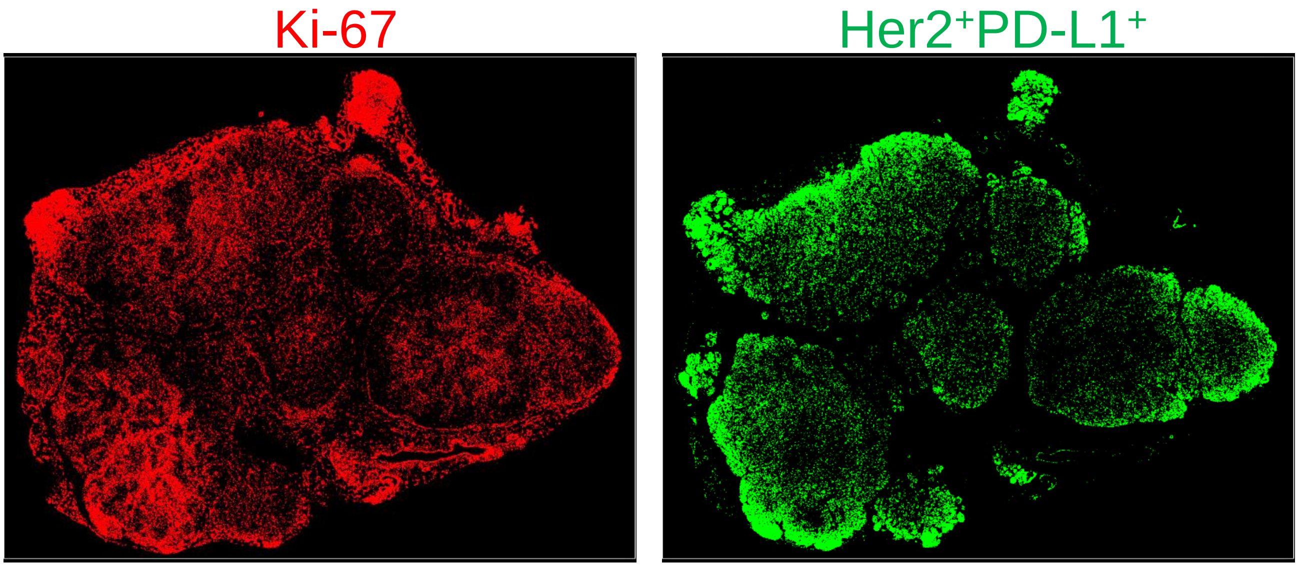

Supplementary Figure S10

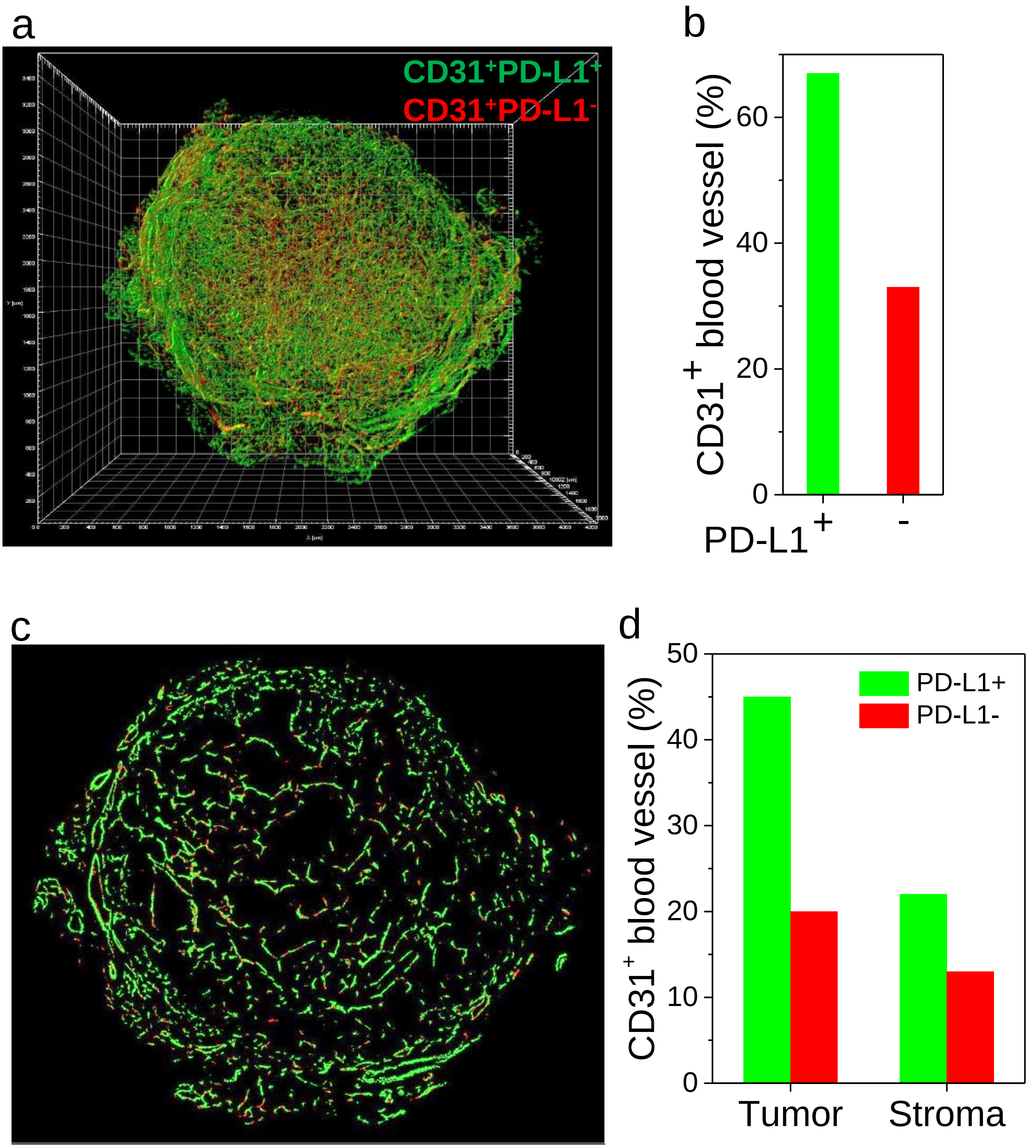

Supplementary Figure S11

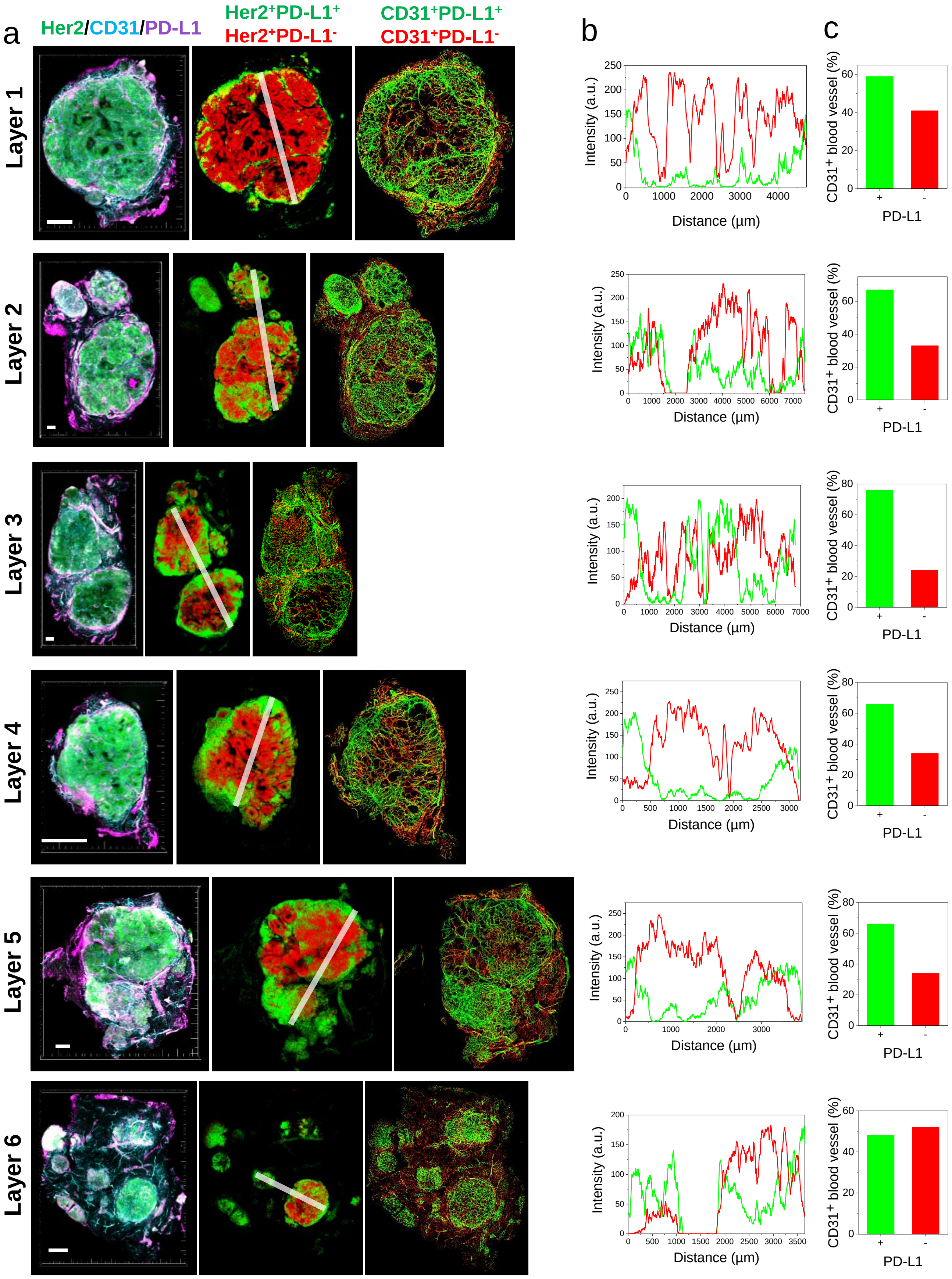

Supplementary Figure S12

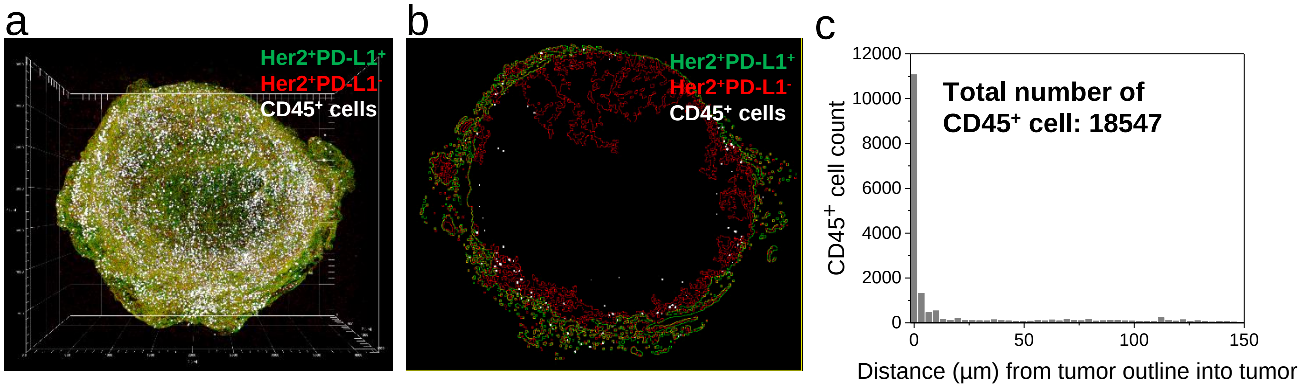

Supplementary Table S1

Antibody	Clone	Source	Antibody Concentration	Fluorescent Dye	Added Dye Solution Volume (at 10 mg/ml DMF*)
Anti-Her2	7.16.4	Antibody Facility at UChicago	0.6 mg/0.5 ml	DyLight^{TM} 488 NHS ester	14 µl
				DyLight^{TM} 633 NHS ester	4.5 µl
Anti-reticular fibroblasts and fibres	ER-TR7	BioXCell	0.5 mg/0.5 ml	DyLight^{TM} 488 NHS ester	14 µl
Anti-CD45	30-F11	Biolegend	0.2 mg/0.4 ml	DyLight^{TM} 550 NHS ester	4 µl
Anti-Ki-67	16A8	Biolegend	0.05 mg/0.1 ml	DyLight^{TM} 594 NHS ester	1 µl
Anti-CD31	MEC13.3	Biolegend	0.2 mg/0.4 ml	DyLight^{TM} 633 NHS ester	3 µl
Anti-PD-L1	10F.9G2	BioXCell	0.6 mg/0.6 ml	DyLight^{TM} 680 NHS ester	11 µl

*DMF: Dimethylformamide

Supplementary Table S2

Macro	Function
LIFtile-restitcher	align and stitch 3D mosaics for multi-channel images (0-999 image tiles)
HPRstack2ConstantMean	compensate for depth-related intensity losses
composite big aligner	automate alignment and registration of the stitched macrosection images
closeZvoids	merged top slice and bottom slice of the macrosections
hyprBKGDfix	clear background around tumor and tissue
wekaMacro	automate segmentation of the cells only showing fluorescence signals on the membrane (e.g. CD45)
vessel extractor	automate segmentation of the $CD31^+$ blood vessels
HER2outlinerMacro	automate segmentation $Her2^+$ tumor outlines

Fiji macro script 1. LIFtile-restitcher

```
/*Macro to apply a common transform to align and stitch 3D mosaics using FIJI Grid/Collection plugin
 * Specific case is for Leica LIF data, using a specific channel as a reference; for BIG DATA!
 * Since bioformats fails to correctly open LIF tile scanning order, this macro requires export to single tifs
 *  and pulls in all stage/CH/Z files for stitching. PREP WORK:
 *                    Open a Leica merge to count up the RxC values used in capture [see note below]
 *                    EXPORT_AS_TIF the LIF file into a folder using Leica software, and create a separate output folder
 *                    Look at last export files to get Z step numbers, BUT, ADD ONE [starts with zero not 1]
 *                    RefCH is main reference for all others --fit performed for RefCH and then same is applied to all other channels
 *                    and then assembles all into a composite stack [if memory allows]
 *                    This macro EDITS the transformation text file to call each channel (so you don't have to manually!)
 *                   You can edit the code below for other stitching options; default is to ignore Z shifts and use random intensity blending [best options!]
 *                                  <v8 bug fixes: input dimensions, plus max projection generation at end
 *                    Code is for current FIJI, all features as default plugins/features
 *                    this pads names for grid stitcher with leading zeros up to 100000 stage locales
 *                   If you didn't note the rowsXcols layout of your data collection, you can look in the Metadata folder
 *                   that LASAF produces when exporting tifs. Find the XML with same name (not the 'properties' one)
 *                   and scroll down to last X and Y physical location lines near top and note last line for X and Y fields
 *                   (add one to each since number from 0)
 *                                 tile alignment default is by location or can ask for fitting (can result in weird results at times)
 *                                 Edge hiding default is random intensity or can do linear fits
 *                                 v12 corrects bug in computed stitching file name v13 allows for single channel input
 *                                 v14 adds field for tile overlap value (can enlarge actual and ask to compute fit)
 *                                 v15 allows for altered collection on SP8 LASX system
 *                                 v16 adds warning comments for LONG code lines [n=3] that might be broken by text editors or email
 *                   note to self-- sp5 MATRIX order seems to be col x col, up+rt, flip row-col vals
 * V.Bindokas, Univ of Chicago, Oct 2017
 */

function leftPad(n, width) {    // pad code from example macros
   str =""+n;
   while (lengthOf(str)<width)
      str = "0"+str;
   return str;
   }

run("Close All");

pv=newArray("sp5", "sp8");
fuz=newArray("rand", "linear");
olp=newArray("compute", "not");
Dialog.create("setup");
 Dialog.addChoice("system", pv, "sp5");
 Dialog.addNumber("tiles across", 5);
 Dialog.addNumber("vertical", 7);
 Dialog.addNumber("tileOverlap%",10);
 Dialog.addNumber("Ch", 4);
 Dialog.addNumber("Z", 3);
 Dialog.addNumber("RefCH", 1);
 Dialog.addChoice("FitMthd", olp, "not");
 Dialog.addChoice("SeamingMthd", fuz, "rand");
 Dialog.show();
 p = Dialog.getChoice() pv;
 Xs = Dialog.getNumber()"Rows";                    //order read is order above!
 Ys = Dialog.getNumber()"Col";
 T = Dialog.getNumber()"tol";
 ch = Dialog.getNumber()"Ch";
 z = Dialog.getNumber()"Z";
 ref= Dialog.getNumber()"RefCH";
 o = Dialog.getChoice() olp;
 fu = Dialog.getChoice() fuz;
 if (fu=="rand") F = "Intensity of random input tile";
 if (fu=="linear") F = "Linear Blending";
 st = Xs * Ys;
 if (p=="sp5") way="Right";
 else {
          way="Left";
          tmp=Xs;                    //lasx swapped rows and col vs lasaf, just to mess with us
          Xs=Ys;
          Ys=tmp;
 }
d1=getDirectory("Choose input Directory");
li=getFileList(d1);
d2=getDirectory("Choose output Directory");
fup=d1+li[1];


//------setup part, copy out data for stitching format-------


for (S=0; S<st; S++){                    //this makes blocks of images the tiler seems to require
                    if (st>10 && S<10) ss="0"+toString(S);          //stringify and pad counter value to match leica naming scheme [number of digits]
                    else ss=toString(S);
                    if (st>100 && S<100 ) ss="0"+ss; //stringify and pad, add another zero for the big-job folks...
                    if (st>1000 && S<1000 ) ss="0"+ss; //stringify and pad, add another zero for the big-job folks...
                    if (st>10000 && S<10000 ) ss="0"+ss; //stringify and pad, add another zero for the big-job folks...

          run("Image Sequence...", "open=[fup] file=_s"+ss+" sort");                    //import all files per stage position
          if (ch>1) run("Stack to Hyperstack...", "order=xyczt(default) channels=&ch slices=&z frames=1 display=Color");
          run("Gaussian Blur...", "sigma=1 stack");                    //denoise all, just a touch
          if (ch>1) run("Split Channels");
          for (h=ch;h>=1;h--){
                    ss=toString(S);
                    ss=leftPad(parseInt(ss),5);                    //good for 100k images;  pad more if you got the RAM
                    pa=d2+"S"+ss+"_C"+h+".tif";
                    saveAs("Tiff", pa);                    //write out data for stitching, keeping memory needs lower
                    close();
          }          //for h
}   //for S

//------end setup part---------

          //recorded stitch setup below doesn't use 'calculation' of fits to avoid the common goof ups dropping things or shifting Z
          //change to whatever you feel like trying. The alignment map is then applied to all other channels vs trying to fit each independently. So, make sure reference CH is a good one!
          //this computes the fitted tile layout with acceptance of any-all quality factor
if (o == "not"){
          //caution: extremely long single line follows, then a "}" as next line
run("Grid/Collection stitching", "type=[Grid: row-by-row] order=["+way+" & Up] grid_size_x=&Xs grid_size_y=&Ys tile_overlap=&T first_file_index_i=0 directory=[&d2] file_names=S{iiiii}_C"+ref+".tif output_textfile_name=TileConfiguration.txt fusion_method=["+F+"] regression_threshold=0.00 max/avg_displacement_threshold=333 absolute_displacement_threshold=422 ignore_z_stage subpixel_accuracy display_fusion computation_parameters=[Save computation time (but use more RAM)] image_output=[Fuse and display]");
}
else {
          //caution: extremely long single line follows, then a "}" as next line
run("Grid/Collection stitching", "type=[Grid: row-by-row] order=["+way+" & Up] grid_size_x=&Xs grid_size_y=&Ys tile_overlap=&T first_file_index_i=0 directory=[&d2] file_names=S{iiiii}_C"+ref+".tif output_textfile_name=TileConfiguration.txt fusion_method=["+F+"] regression_threshold=0.00 max/avg_displacement_threshold=333 absolute_displacement_threshold=422 compute_overlap ignore_z_stage subpixel_accuracy display_fusion computation_parameters=[Save computation time (but use more RAM)] image_output=[Fuse
and display]");
}

pa="fused_ch"+ref+".tif";
saveAs("Tiff", d2+pa);
close();



dx = newArray("1");                    //build array for doing other channels
if (ch>1){
          for (dxi=2; dxi<=ch; dxi++){
                    dx=Array.concat(dx, dxi);
          }

          for (i=0;i<ch; i++){                    // BE AWARE array index starts as ZERO not 1
                    if ( parseInt(dx[i]) != ref){
                              A="_C"+toString(ref);
                              B="_C"+toString(parseInt(dx[i]));
                              nu=d2+"next.txt";                    //register data map with next CH in names vs CH<ref> of 1st run
                              if (o == "not")  {          fi=d2+"TileConfiguration.txt";}                    //the plain fits are named thusly
                              else fi=d2+"TileConfiguration.registered.txt";                    //the computed fits are named thusly
                              str=File.openAsString(fi);
                              rows=split(str, "\n");
                              x=newArray(rows.length);
                                        for(k=0; k<rows.length; k++){                    //do the filename string edits for new input
                                                  rows[k]=replace(rows[k], A, B);
                                                  print(rows[k]);
                                                  File.append(rows[k], nu);
                                                  File.append("\r", nu);
                                        }
                              //apply stitiching and write result to disk
                    //caution: extremely long single line follows, then next line starts "pa="
                              run("Grid/Collection stitching", "type=[Positions from file] order=[Defined by TileConfiguration] directory=[&d2] layout_file=next.txt fusion_method=["+F+"] regression_threshold=0.00 max/avg_displacement_threshold=333 absolute_displacement_threshold=433 ignore_z_stage subpixel_accuracy display_fusion computation_parameters=[Save computation time (but use more RAM)] image_output=[Fuse and display]");
                              pa="fused_ch"+(dx[i])+".tif";
                              saveAs("Tiff", d2+pa);
                              File.delete(nu);
                              close();
                    }          //end if not ref CH
          }          //end for all CH
}          //end ch>1

//pull all CH output back in for building the large composite
for (i=1;i<=ch; i++){
          run("Bio-Formats Importer", "open=["+d2+"fused_ch"+i+".tif] color_mode=Default view=Hyperstack stack_order=XYCZT");          //virtual stack has bug--- not doing Z!
}
Stack.getDimensions(width, height, channels, slices, frames);
run("Concatenate...", "all_open title=all");
run("Stack to Hyperstack...", "order=xyzct channels=&ch slices=&slices frames=1 display=Composite");  //you can add steps to set colors specifically after this line, if you need to
pa="fused_merged.tif";
saveAs("Tiff", d2+pa);
if (ch>1){
          run("Z Project...", "projection=[Max Intensity]");
          pa="MAXfused_merged.tif";
          saveAs("Tiff", d2+pa);
}
```

Fiji macro script 2. HPRstack2ConstantMean

```
//macro to normalize mean value per slice to that of average per channel
// hyperstacks version; this bases slice mean on thresholded value vs all image
// V.Bindokas, Univ of Chicago, AUG 2015

run("Set Measurements...", "mean standard modal min median limit redirect=None decimal=3");
ti=getTitle();
Stack.getDimensions(w, h, ch, sl, fr);
run("Gaussian Blur...", "sigma=1 stack");                    //denoise all, just a touch
end=sl/2;                    //use 1st half of confocal data for measures (since deep layers are fainter)
run("Z Project...", "stop=&end projection=[Average Intensity]");
av=getImageID();
if (Stack.isHyperstack==1) Stack.setDisplayMode("color");      //test so this can run on non-hyperstacks too

selectImage(ti);
if (Stack.isHyperstack==1) Stack.setDisplayMode("color");

mean=0;
min=0;
for (i=1; i<=ch; i++){
                    //find signal avg intensity
                    run("Clear Results");
                    selectImage(av);
                    setSlice(i);
                    run("Select None");
                    setAutoThreshold("Otsu dark");
                    run("Create Selection");
                    List.setMeasurements;
  mean = List.getValue("Mean");
  median = List.getValue("Median");
                    run("Make Inverse");          //measure background
                    List.setMeasurements;
  min = List.getValue("Mean");

for (j=1; j<=sl; j++){
                     selectWindow(ti);
                     Stack.setPosition(i, j, 1);      //format-- channel, slice, frame
                     run("Subtract...", "value=&min slice");                //subtract avg minimum
                     run("Select None");
                     setAutoThreshold("Otsu dark");
                     run("Measure");                                       //this is faster vs creating a selection
                     jmean = getResult("Mean", nResults-1);                //get current mean
                     jmdn = getResult("Median", nResults-1);               //get current median
                     adj=mean/jmean;
                     print(mean, j, jmean, adj, jmdn);
                     run("Select None");
                     run("Multiply...", "value=&adj slice" );              //correct the mean
    }//for j slices
}//for all i CH

selectImage(av);
close();
selectWindow(ti);
if (Stack.isHyperstack==1) Stack.setDisplayMode("composite");
Stack.setPosition(1, 1, 1);
rename("nrmlzd2-"+ti);
```

Fiji macro script 3. composite big aligner

```
/*Make sure no images are open on launching this, except the big hyperstack to align
 * this vers aligns layer n+1 to layer n
 *
 * this requires download+install of multistackreg plugin:
 * http://bradbusse.net/MultiStackReg1.45_.jar
 * V.Bindokas, Univ of Chicago, Aug 2015
*/



Dialog.create("tell me");
Dialog.addNumber("number of layers:", 2);
Dialog.show();
la = Dialog.getNumber();

waitForUser("scroll to the 1st slice of layer 2, then hit OK");
ti=getTitle();
i0=getImageID();
selectImage(i0);
getDimensions(w, h, ch, sl, fr);                    //measure each for resizing to common W H
Stack.getPosition(c1, s1, f1);

run("Z Project...", "stop=&s1 projection=[Average Intensity]");          //layer projection
if (ch>1){
                zp=getImageID();
                run("Z Project...", "projection=[Average Intensity]");          //in case of hyperstack
                selectImage(zp);
                close();
}

li=newArray("1");
lim=newArray();
for (k=1;k<=la;k++){                                   //populate an array to hold layer limits
                lim=Array.concat(lim,li);
}
lim[1]=s1;

for (L=2; L<la; L++){
                selectImage(i0);
                waitForUser("scroll to 1st slice of next layer, then hit OK");
                Stack.getPosition(c, s, f);
                run("Z Project...", "start=&s1 stop=&s projection=[Average Intensity]"); //layer projection
                if (ch>1){
                                zst=getImageID();
                                run("Z Project...", "projection=[Average Intensity]");          //in case of hyperstack
                                selectImage(zst);
                                close();
                }

                lim[L]= s;
                s1=s;                           //set for next layer
                }

Array.print(lim);

//last layer
selectImage(i0);
run("Z Project...", "start=&s1 stop=&sl projection=[Average Intensity]");  //project what's left
                if (ch>1){
                                zst=getImageID();
                                run("Z Project...", "projection=[Average Intensity]");          //max of max hyperstack
                                selectImage(zst);
                                close();
                }


run("Images to Stack");                                //this is why no other images can be open on launching....
rename("ref");

setBatchMode(true);

selectImage(i0);

                //##### fix layer2 onwards ######

for (l=1; l<la; l++) {                          //for all layer slices; will need to run one more time for last layer after this!
                                selectWindow("ref");
                                setSlice(l);
                                run("Duplicate...", "title=this");
                                selectWindow("ref");
                                setSlice(l+1);
                                run("Duplicate...", "title=nxt");
                                run("MultiStackReg", "stack_1=this action_1=[Use as Reference] file_1=[] stack_2=nxt action_2=[Align to First Stack] file_2=tform transformation=[Rigid Body] save");
                                selectWindow("this");
                                close();
                                selectWindow("nxt");            //replace ref with aligned vers
                                run("Copy");
                                close();
                                selectWindow("ref");
                                run("Paste");
                                selectImage(i0);
                                strt=lim[l];                    //retrieve layer range from array. Since arrays index from 0, this is range for layer NEXT
                                if (l+1 < lim.length) stop=lim[l+1]-1;
                                else stop=sl;

print(l,strt,stop);

                                for (sli=strt; sli<=stop; sli++){
                                                selectImage(i0);
                                                for (lac=1; lac<=ch; lac++) {
                                                                Stack.setPosition(lac, sli, f1);
                                                                run("Duplicate...", "duplicate channels=&lac slices=&sli");
                                                                rename("wrk");
                                                                run("MultiStackReg", "stack_1=wrk action_1=[Load Transformation File] file_1=tform stack_2=None action_2=Ignore file_2=[] transformation=[Rigid Body]");
                                                                selectWindow("wrk");
                                                                run("Copy");
                                                                close();
                                                                selectImage(i0);
                                                                run("Paste");
                                                }//end for CH
                                }//end for sli
}//end l --for all layers

rename("aligned-"+ti);
selectWindow("ref");
close();
setBatchMode("exit and display");
```

Fiji macro script 4. closeZvoids

```
/* FIJI macro to close up Z voids by means of MAX projections
 *  merges previous slice with current, deletes previous
 *            input: multiCH hyperstack or regular stack
 *                        position stack above the slice to remove before running this
 *  V.Bindokas, UChicago, JUL 2015
 */

ti=getTitle();
Stack.getDimensions(w, h, c, s, f);
Stack.getPosition(ch, sl, fr);
if (c>1){
            run("Duplicate...", "title=now duplicate slices="+sl);
            selectWindow(ti);
            run("Duplicate...", "title=pre duplicate slices="+sl-1);
            imageCalculator("Max stack", "now","pre");                        //overwrite 'now' with max
            imageCalculator("Average create stack", "now","pre");
            imageCalculator("Average stack", "now","Result of now");          //weigh MAX projection with AVG, overwriting 'now'
            selectWindow("Result of now");
            close();                                                          //doing just multiple AVG reductions with dark areas decr signal too much, and MAX alone becomes too noisy
            selectWindow("pre");
            close();
            selectWindow("now");
            ns=nSlices;
            for (i=1;i<=ns; i++){
                        selectWindow("now");
                        setSlice(i);
                        run("Select All");
                        run("Copy");
                        selectWindow(ti);
                        Stack.setPosition(i, sl, fr);
                        run("Paste");
            }

            selectWindow(ti);
            Stack.setPosition(ch, sl-1, fr);
            run("Delete Slice", "delete=slice");
            Stack.setPosition(ch, sl, fr);            //set to next slice (now one less)
            selectWindow("now");
            close();
            run("Collect Garbage");                                           //this line is fiji, will need to commented out if ImageJ
}
else {
            run("Duplicate...", "title=now");
            selectWindow(ti);
            Stack.setPosition(ch, sl-1, fr);
            run("Duplicate...", "title=pre");
            imageCalculator("Max stack", "now","pre");
            selectWindow("pre");
            close();
            selectWindow("now");
            run("Select All");
            run("Copy");
            close();
            selectWindow(ti);
            Stack.setPosition(ch, sl, fr);
            run("Paste");
            selectWindow(ti);
            Stack.setPosition(ch, sl-1, fr);
            run("Delete Slice", "delete=slice");
            Stack.setPosition(ch, sl, fr);            //set to next slice (now one less)
            run("Collect Garbage");                          //this line is fiji, will need to commented out if ImageJ
}
```

Fiji macro script 5. hyprBKGDfix

```
/* macro to clear background around tissue
 *  input is multichannel Zstack. this runs forward from the slice set at start!
 *  V.Bindokas, Univ of Chicago, SEPT 2015
 */
run("Colors...", "foreground=white background=black selection=yellow");
run("Set Measurements...", "area mean standard modal min integrated redirect=None decimal=3");
ti=getTitle();
Stack.getDimensions(w, h, c, s, f);
Stack.getPosition(ch, sl, fr);

if (c>1){                               //routine needs composite view mode to extract all CH vs single CH
        Stack.getDisplayMode(mode);
        if (mode =="color") {Stack.setDisplayMode("composite");}
}

setBatchMode(true);
for (p=sl; p<=s; p++){
        Stack.setPosition(1, p, fr);

        if (c>1){
                run("Duplicate...", "title=wrk");
                ns=nSlices;
                selectWindow("wrk");
                run("Z Project...", "projection=[Median]");          //change projection and try again
                setAutoThreshold("Li dark");                          //threshold method- change as needed
                getThreshold(lo, up);
                resetThreshold();
                setThreshold(lo, up);
                run("Create Selection");
                close();
                selectWindow("wrk");
                run("Restore Selection");
                run("Enlarge...", "enlarge=-1");     // to smooth ROI, drop single pixels
                run("Enlarge...", "enlarge=1");
                run("Clear Outside");

                for (i=1;i<=ns; i++){
                        selectWindow("wrk");
                        setSlice(i);
                        run("Select All");
                        run("Copy");
                        selectWindow(ti);
                        Stack.setPosition(i, p, fr);
                        run("Paste");
                }
                selectWindow("wrk");
                close();
                run("Collect Garbage");
        }
}
rename("cleaned_"+ti);
```

Fiji macro script 6. wekaMacro

```
/*WEKA object extractor macro for FIJI
 * This needs you to create and save a classifier prior to running
 * WEKA can take a LONG time and esp for large data volumes
 *         ___Be sure to CHANGE the file locations hard-coded in the lines 21 and 34___
 * V.Bindokas, Univ of Chicago, Nov 2015
 */


ti=getTitle;
ns=nSlices();

for (i=1; i<=ns; i++){
		selectWindow(ti);
		setSlice(i);
		run("Duplicate...", "title=wrk");
		setTool("freeline");
		run("Trainable Weka Segmentation");
		wait(50);				//add delays to work around random stalls...
		selectWindow("Trainable Weka Segmentation v2.3.0");
		wait(50);
		//_______________CHANGE NEXT LINE TO YOUR CLASSIFIER LOCATION!!!________________
		call("trainableSegmentation.Weka_Segmentation.loadClassifier", "E:\\Image data\\bio_CD3_CD8_CD31\\Classification\\CD3_classifier_3.model");
		wait(50);
		call("trainableSegmentation.Weka_Segmentation.getResult");
		while (isOpen("Classified image")==false){
				wait(10000); print("waiting");
		}
		selectWindow("Classified image");
		run("Grays");
		setThreshold(0, 0);
		setOption("BlackBackground", true);
		run("Convert to Mask");
		run("Median...", "radius=2");
		//_______________CHANGE NEXT LINE TO YOUR SELECED OUTPUT LOCATION!!!________________
		run("Save", "save=[E:\\Image data\\bio_CD3_CD8_CD31\\Classification\\Bio1_2\\CD3 class\\CLimge "+i+".tif]");
		selectWindow("Trainable Weka Segmentation v2.3.0");
		wait(50);
		close();
		selectWindow("wrk");
		wait(50);
		close();
		selectWindow("CLimge "+i+".tif");
		wait(50);
		close();
		run("Collect Garbage");					//comment this line out if not using FIJI
}
```

Fiji macro script 7. vessel extractor

```
/*CD31 vessel extractor
 * input is grayscale tile fusion for CH3 preprocessed with stack2ConstantMean_1CH
 * V.Bindokas, Univ of Chicago, MAY 2015 rev JUN 2017 with alt filters
 */
run("Set Measurements...", "area mean standard redirect=None decimal=3");
run("Colors...", "foreground=white background=black selection=yellow");
ti=getTitle();
run("Select None");
getVoxelSize(width, height, depth, unit);
Stack.getDimensions(w, h, ch, sl, fr);
if (Stack.isHyperstack) {
		Stack.getDisplayMode(mode);
			if (mode == "composite") Stack.setDisplayMode("color");

		//threshold only works in non-composite mode
}

newImage("vasc", "8-bit black", w, h, sl);
setVoxelSize(width, height, depth, unit);

selectImage(ti);

run("Subtract Background...", "rolling=4 sliding stack");
selectWindow(ti);
run("Gaussian Blur 3D...", "x=.75 y=.75 z=.75");			//this is a 3D unsharp mask processing
run("Duplicate...", "title=blr duplicate");			//this is a 3D unsharp mask processing
run("Gaussian Blur 3D...", "x=9 y=9 z=1");			//this is a 3D unsharp mask processing
imageCalculator("Subtract stack", ti,"blr");			//this is a 3D unsharp mask processing
run("Gaussian Blur 3D...", "x=.75 y=.75 z=.75");
setAutoThreshold("Triangle dark stack");
getThreshold(lo, upper);
up=64095;			//for our 12bit images... need high value if-for deconvolved images
selectWindow("blr");
close();

setBatchMode(true);


for (s=1; s<=sl; s++){
		selectImage(ti);
		Stack.setSlice(s);
		selectImage(ti);
		run("Duplicate...", "title=slc  slices="+s);
		setThreshold(lo, up);
//note the size range is MICRONS by intent, not PIXELS, so make sure calibration is ok
		run("Analyze Particles...", "size=10-Infinity circularity=0.00-0.95 show=Masks");
		run("Grays");
		selectWindow("Mask of slc");
		run("Select All");
		run("Copy");
			selectWindow("vasc");
			Stack.setSlice(s);
			run("Paste");
			selectWindow("slc");
			close();
			selectWindow("Mask of slc");
			close();
			run("Collect Garbage");
}
run("Minimum 3D...", "x=0 y=0 z=2");  //this trims Z stretch and removes some small junk
selectWindow("vasc");
rename("vasc-v6-"+ti);
selectImage(ti);
close();
```

Fiji macro script 8. HER2outlinerMacro

```
/*HER2 outliner
 * input is 16bit single CH stack
 * V.Bindokas, Univ of Chicago, DEC 2015
 */
run("Colors...", "foreground=white background=black selection=yellow");
siz=20;			//min object size in pixels
ti=getTitle();
Stack.getDimensions(w, h, c, s, f);
Stack.getPosition(ch, sl, fr);
setBatchMode(true);
for (i=1;i<=s; i++){
			selectWindow(ti);
			setSlice(i);
			run("Select None");
			resetThreshold();
			setAutoThreshold("Otsu dark");
			run("Analyze Particles...", "size=&siz-infinity pixel show=Masks slice");
			run("Grays");
			run("Options...", "iterations=3 count=1 black do=Nothing");
			setOption("BlackBackground", true);
			run("Dilate");				//close small voids
			run("Erode");
			run("Outline");
			run("Select All");
			run("Copy");
			selectWindow(ti);
			run("Paste");
			selectWindow("Mask of "+ti);
			close();
			run("Select None");
}
resetThreshold();
run("8-bit");
run("Multiply...", "value=300 stack");
rename("edges-"+ti);
```
